# α-Amylase: Its Structure, Molecular Modification, and Application in the Food Field

**DOI:** 10.3390/foods15091555

**Published:** 2026-04-30

**Authors:** Gang Liu, Manuel Montalbán-López, Dehua Wei, Lei Wang, Xuefeng Wu, Xingjiang Li, Dongdong Mu

**Affiliations:** 1 Anhui Fermented Food Engineering Research Center, Key Laboratory for Agricultural Products Processing of Anhui Province, School of Food and Biological Engineering, Hefei University of Technology, Hefei 230009, China; 2024171638@mail.hfut.edu.cn (G.L.); 13849961172@163.com (D.W.); 2025020133@mail.hfut.edu.cn (L.W.);; 2 Institute of Biotechnology, Department of Microbiology, Faculty of Sciences, University of Granada, 18071 Granada, Spain; manuelml@ugr.es; 3 Division of Life Sciences and Medicine, The First Affiliated Hospital of University of Science and Technology of China, University of Science and Technology of China, Hefei 230001, China

**Keywords:** α-amylase, protein engineering, structure determination

## Abstract

This review comprehensively examines the structural architecture, catalytic mechanisms, and targeted molecular engineering of α-amylase (primarily the GH13 family), a pivotal biocatalyst in the food industry. We highlight diverse microbial sources of α-amylases and their cost-effective heterologous expression in well-characterized hosts like *Bacillus subtilis* and *Escherichia coli*. To overcome extreme operational bottlenecks—such as elevated temperatures and acidic environments—recent advances in protein engineering are critically evaluated. These strategies, including directed evolution, semi-rational design, and advanced immobilization on nanomaterials, synergistically enhance the enzyme’s thermostability, catalytic efficiency, and reusability. Furthermore, this paper synthesizes the state-of-the-art applications of engineered α-amylases across key food processing sectors, including baking, sugar refining, and brewing. By integrating structural biology with advanced material science, this review provides a targeted roadmap for developing next-generation, high-performance α-amylases to address current and future challenges in sustainable food processing.

## 1. Introduction

Starch is a major component of the human diet, with corn, wheat, rice, potato, and cassava serving as its primary sources. This widely used and versatile polysaccharide is not only directly present in the diet but also utilized as a raw material for the manufacture of starch-based products, modified starches, and other derivatives across the food, textile, cosmetic, and pharmaceutical industries. Starch is composed exclusively of glucose molecules linked by glycosidic bonds to form two distinct polymers: linear amylose (linked by α-1,4-D-glucosidic bonds) and branched amylopectin (containing both α-1,4-D- and α-1,6-D-glucosidic linkages). Starch can be hydrolyzed into glucose, maltose, maltodextrin, and other oligosaccharides by different enzymes generally referred to as amylases, which can be classified as exoamylases and endoamylases, according to their mode of action [1]. Exoamylases, including β-amylase (EC 3.2.1.2) and glucoamylase (EC 3.2.1.3), systematically hydrolyze α-1,4-glycosidic bonds from the non-reducing ends of starch to yield maltose and glucose, respectively. Conversely, endoamylases, such as α-amylase (EC 3.2.1.1), randomly cleave internal α-1,4-linkages, degrading starch primarily into dextrins and malto-oligosaccharides. However, to transcend this traditional functional division, the Carbohydrate-Active enZymes (CAZy) database (http://www.cazy.org/, accessed on 27 March 2026) provides a more rigorous structural classification framework [2]. Within this system, these three principal amylolytic enzymes are assigned to distinct glycoside hydrolase (GH) families: α-amylase predominantly belongs to the GH13 family, β-amylase to the GH14 family, and glucoamylase to the GH15 family. It is imperative to note that although these three enzymes share starch as a common substrate, they possess no evolutionary correlation and represent independently evolved, distinct catalytic machineries. Specifically, they lack homology in their amino acid sequences. Regarding catalytic domain folds, GH13 features a classic (β/α)8-barrel fold, GH14 contains a supersecondary structure with unique long loop insertions, while GH15 exhibits a completely distinct (α/α)6-barrel funnel configuration. Furthermore, their catalytic machineries are vastly different; GH13 relies on a classical catalytic triad composed of aspartate and glutamate (Asp-Glu-Asp), whereas GH14 and GH15 predominantly depend on a catalytic dyad formed by a pair of glutamate (Glu) residues. In terms of reaction mechanisms, GH13 undergoes substrate cleavage via a double-displacement retaining mechanism that preserves the α-configuration at the anomeric carbon of the product. Conversely, GH14 and GH15 facilitate direct attack by a water molecule via a single-displacement inverting mechanism, yielding a product with an inverted β-configuration [1,3,4]. Adopting this classification based on sequence and core structure can more accurately elucidate the structure–function relationships of the enzymes, thereby thoroughly avoiding potential confusion caused by superficial nomenclature [5].

They are used in grain processing, the food industry, brewing, fermentation, textiles, medicine, and oil extraction, thus becoming one of the most relevant enzymes in industry. α-amylase is an endoactive amylase that can cleave starch under neutral pH conditions, thus making the viscous starch paste quickly lose viscosity and liquefy. In these conditions, the iodine coloring reaction disappears very quickly, so it is also known as a starch liquefying enzyme. The α-amylase is widely applied in various fields of the food industry. For example, besides its role in starch liquefaction and saccharification, α-amylase is further utilized in textile sizing, juice and beer clarification, feed manufacturing, detergent manufacturing, biofuel production, and other industrial applications. Nevertheless, wild-type α-amylases exhibit suboptimal performance under the extreme conditions encountered in certain industrial processes; thus, molecular modifications are required to enhance their robustness in such harsh environments [6]. While structure-based classification provides a fundamental framework, the industrial realization of α-amylases relies heavily on overcoming operational bottlenecks, particularly their insufficient thermal stability under harsh processing conditions.

To address these challenges, this paper reviews the fundamental kinetics and catalytic mechanisms of α-amylases, alongside advanced enzyme immobilization strategies and targeted molecular modifications. By systematically evaluating how these approaches enhance structural rigidity and overall catalytic efficiency, this review establishes a crucial theoretical and practical foundation for their extensive application in the food industry.

While the last decade has seen numerous reviews on α-amylase, the rapid convergence of computational biology and advanced material science necessitates an updated, highly focused synthesis. Unlike previous generalized overviews, this review explicitly bridges the gap between modern AI-driven structural predictions and contemporary semi-rational design paradigms. Furthermore, we critically assess how these targeted molecular modifications, synergistically coupled with cutting-edge immobilization techniques (such as nanoconfinement and metal–organic frameworks), specifically address the extreme operational bottlenecks of the modern food processing industry. By integrating structural flexibility engineering with advanced carrier rigidification, this paper provides a uniquely targeted and actionable roadmap for engineering next-generation, high-performance α-amylases tailored for harsh industrial environments.

## 2. Sequence, Structure and Catalytic Mechanism of α-Amylases

### 2.1. Sequence Classifications of α-Amylase

Based on the primary structural similarity of their catalytic domains, the vast majority of industrially relevant α-amylases are classified into the Glycoside Hydrolase family 13 (GH13) within the CAZy database, with only a minority belonging to families such as GH57, GH119, and GH126. Because GH13 is an exceptionally large and polyspecific family representing approximately 30 different reaction and product specificities—encompassing glycoside hydrolases (EC 3.2.1.x), glucosyltransferases (EC 2.4.1.x and EC 2.4.99.16), and isomerases (EC 5.4.99.x) that all share a conserved structural scaffold—it has been systematically divided into distinct subfamilies [7].

Paying particular attention to these individual GH13 subfamilies is crucial for distinguishing α-amylases from various sources. For instance, the highly thermostable “liquefying” α-amylases from the *Bacillus* genus (e.g., *Bacillus licheniformis* and *Geobacillus stearothermophilus*) predominantly cluster into subfamily GH13_5, whereas the “saccharifying” *Bacillus* α-amylases are typically found in GH13_6. Conversely, fungal α-amylases (such as those from *Aspergillus* species), which are widely used in baking and brewing, are primarily classified into subfamilies like GH13_1 and GH13_15 [8].

**Figure 1:**
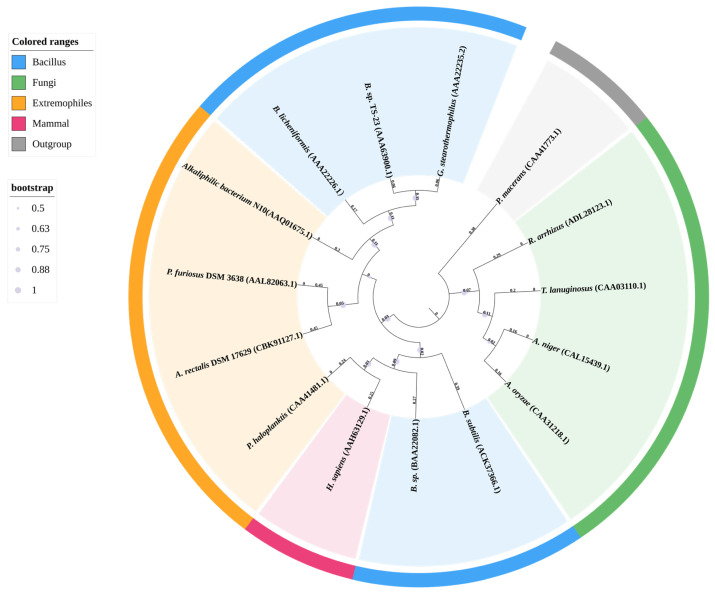
Phylogenetic analysis of representative α-amylases from the GH13 family. The phylogenetic tree was constructed based on the amino acid sequences of mature catalytic domains using the Neighbor-Joining method in MEGA software (version 7.0) and rendered via the iTOL online tool (version 6.0). Sequences were strategically selected to represent major taxonomic domains (bacteria, archaea, fungi, and mammals) and extreme environmental adaptations. A cyclomaltodextrin glucanotransferase (CGTase) from *Paenibacillus macerans* (CAA41773.1) was used as the outgroup to root the tree. The outer colored rings represent specific taxonomic or ecological groupings: blue for the Bacillus group, green for fungi, orange for extremophiles, pink for mammals, and grey for the outgroup. GenBank accession numbers are indicated in parentheses after the species names.

To illustrate this evolutionary diversity and highlight enzymes of industrial significance, we constructed a phylogenetic tree focusing explicitly on a representative set of GH13 α-amylases (EC 3.2.1.1). Rather than an exhaustive display, sequences were strategically selected to encompass major taxonomic domains, reflect their respective GH13 subfamilies, and represent the extreme environmental adaptations and key industrial strains discussed in this review. This approach reveals the evolutionary relationships most relevant to modern protein engineering (Figure 1).

### 2.2. Structure of α-Amylase

**Figure 2:**
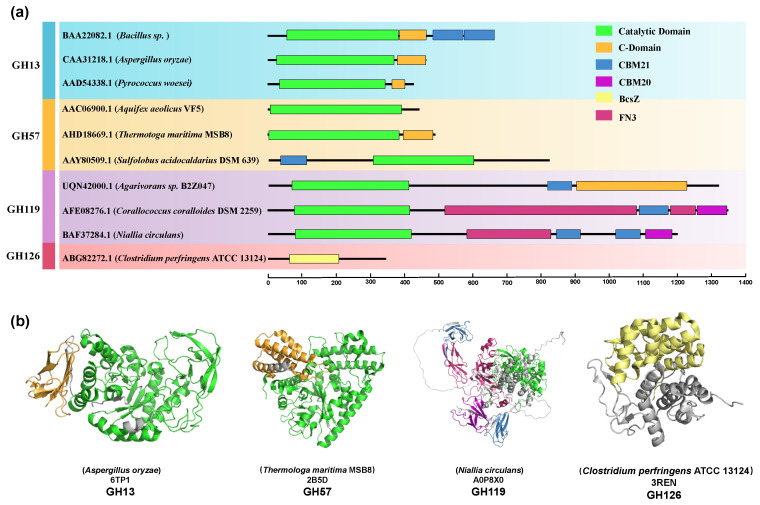
(**a**) Conserved α-amylase domains. The conserved domains were identified using the conserved domain database (http://www.ncbi.nlm.nih.gov/Structure/cdd/cdd.shtml, accessed on 17 March 2026). The green region represents the catalytic domain, the orange region represents the C-terminal domain, the blue region and purple region represent the Carbohydrate binding domain, the red region represents the Fibronectin type 3 domain, and the yellow region represents the Endo-1,4-beta-D-glucanase Y. (**b**) Comparison of the domains of different α-amylase families. The color code of the domain is consistent with the domain organization overview. Visualization of structure by PyMOL software (version 3.0).

To elucidate the intricate diversity inherent in the domain architectures of α-amylases, we systematically selected a set of representative enzymes, which served as the basis for a comprehensive domain analysis aiming to delineate conserved and variable structural elements across this enzyme family (Figure 2). However, to preclude erroneous sequence alignments, the structural analysis of α-amylases must adhere to their specific hierarchical frameworks, as these enzymes span evolutionarily distinct, non-homologous Glycoside Hydrolase (GH) families (GH13, GH57, GH119, and GH126). According to the CAZy classification, families GH13, GH119, and GH126 constitute Clan GH-H, defined by a canonical (β/α)8-barrel fold, whereas family GH57 belongs to Clan GH-T, featuring an incomplete (β/α)7-barrel. Furthermore, recent taxonomic subdivisions of the highly diverse GH57 family restrict true α-amylase specificity exclusively to the GH57_3 subfamily [9]. Additionally, it is worth noting that most carbohydrate-active enzymes are modular proteins; in addition to their catalytic domains, they contain additional modules that play crucial roles in their evolution and function [10]. Carbohydrate binding module (CBM), as a kind of functional auxiliary module, is composed of 40–200 amino acid residues. When these modules specifically target starch or glycogen, they are classically designated as Starch-Binding Domains (SBDs). According to the comprehensive CAZy classification, SBDs do not constitute a single structural homology group but are remarkably diverse, being distributed across at least 15 distinct CBM families (including CBM20, CBM21, CBM25, CBM26, CBM34, CBM41, CBM45, CBM48, CBM53, CBM58, CBM68, CBM69, CBM74, CBM82, and CBM83). Despite this evolutionary diversity, the majority of these SBDs fold into a common β-sandwich structure and frequently possess multiple binding sites that act cooperatively. Their core biological function is to disrupt the surface of raw, insoluble starch granules, thereby enhancing the specific binding affinity and guiding the catalytic domain to the substrate to dramatically improve hydrolysis efficiency [11,12]. The carbohydrate-binding modules (CBMs) of the Amy13K α-amylase confer substrate specificity by enabling high-affinity binding to corn starch granules, thereby dramatically enhancing catalytic efficiency and providing a molecular rationale for the selective utilization of corn-based over potato-based starches by *Eubacterium rectale* in the human gut [13]. The C-terminal domain is speculated to be involved in starch binding, which may enhance the thermal stability of α-amylase through conformational rigidification [14]. The exact function of Fibronectin type 3 domain in bacterial hydrolases is not clear yet. The general assumption supported by experimental data and large-scale bioinformatics analysis is that it may serve as a spatially stable linker between the catalytic domain and CBM, providing the optimal binding location for insoluble substrates such as chitin, cellulose and starch [15]. Despite their benefits in facilitating insoluble substrate degradation, the artificial grafting of CBMs or linkers often imposes unpredictable steric hindrance or thermodynamic instability, remaining a significant bottleneck in chimeric enzyme engineering. Notably, the protein sequences of GH126 family amylases are considerably shorter and share low similarity, with no known carbohydrate-binding modules (CBMs) or other domains in their sequences [16], and no catalytic or active site residues have been identified to date [17]. This intricate interplay of catalytic and non-catalytic domains underscores that the specific domain composition of an α-amylase is fundamental to its functional specialization and evolutionary success.

The advent of high-throughput genome sequencing has unveiled a vast repository of genes encoding microbial amylases, while recent developments in artificial intelligence (AI) have provided a powerful basis for predicting their corresponding protein model structures. This wealth of sequence data provides an indispensable foundation for structural biology. By utilizing these genetic blueprints, scientists can now accurately determine and computationally model the three-dimensional configurations of these enzymes, which is pivotal for elucidating their catalytic mechanisms and guiding rational protein design. Nevertheless, a critical evaluation of AI-driven structural prediction reveals inherent limitations: while these algorithms excel at generating static apo-structures, they remain inadequate for capturing the dynamic conformational transitions induced by substrate binding, as well as the precise coordination spheres of essential metal cofactors (e.g., Ca^2+^), both of which are requisite for rigorous mechanistic characterization.

**Figure 3:**
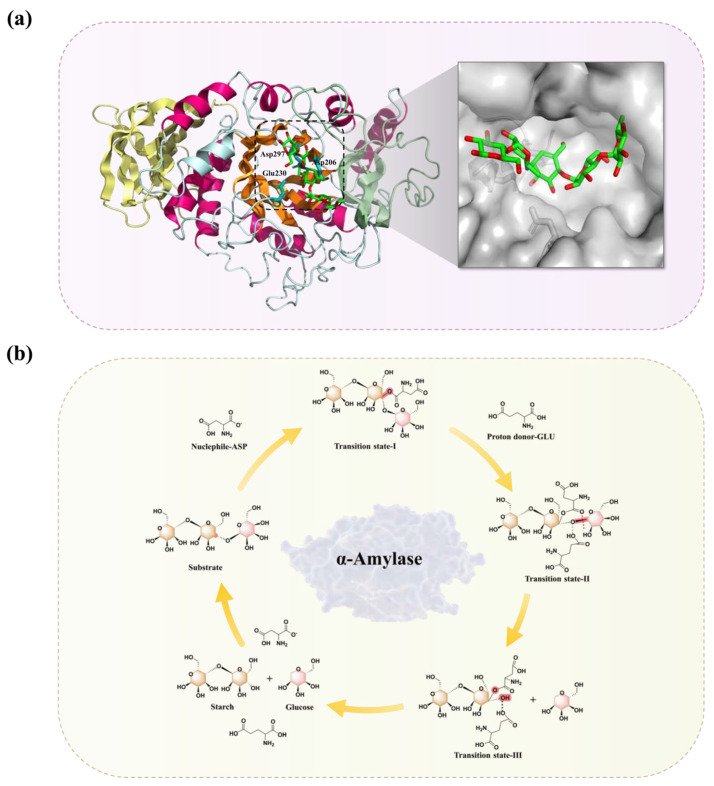
(**a**) Overall structure of Taka-amylase A from Aspergillus oryzae (PDB ID: 2TAA) highlighting the central TIM barrel. The catalytic triad (Asp206, Glu230, and Asp297) is shown as cyan sticks in the active site. The zoomed-in box details the substrate-binding pocket displayed as a gray surface model, with a docked oligosaccharide proxy represented by green and red sticks. The 8 parallel β-strands in the center of the TIM barrel are colored in orange, surrounded by α-helices shown in hot pink. The other distinct structural domains are depicted in pale yellow and pale green, while the remaining secondary structures and connecting loops are in pale cyan. The visualization of the structure was realized by PyMOL software (version 3.0). (**b**) The retaining catalytic mechanism of GH13 α-amylases. The colors are used to distinguish the glucose units during the catalytic cycle; specifically, the red color highlights the terminal glucose molecule that is cleaved and released upon hydrolysis, distinguishing it from the rest of the starch chain (colored in pale yellow).

The majority of Clan GH-H α-amylases have a typical (β/α)8-barrel containing two aspartic acid (Asp) residues and one glutamic acid (Glu) residue, which play pivotal roles in catalysis. The crystal structure of Taka α-amylase A from *Aspergillus oryzae* (TAA) (PDB ID: 2TAA), a representative GH13 member, was the first experimentally determined three-dimensional (3D) structure of an α-amylase, and the (β/α)8-barrel was also the first proved catalytic structural domain of TAA [18] (Figure 3a). Specifically, in TAA, this catalytic triad consists of Asp206, Glu230, and Asp297, which are located at the C-terminal ends of the β-strands. The structural domain A of TAA contains the typical (β/α)8-barrel, also known as the triosephosphate isomerase (TIM) barrel structure, consisting of eight parallel β-strands surrounded by eight α-helices. It serves as the catalytic domain in the α-amylase family. Domain B is located between the third β-strand and the third α-helix of the (β/α)8-barrel, which is mainly involved in substrate or Ca^2+^ binding. The calcium ion establishes interactions with several amino acids contributing to maintain the overall structure, making Domain B a highly sensitive region where minor mutations can inadvertently trigger structural collapse. Last, domain C is formed by eight antiparallel β-strands that are linked to structural domain A by loops. The function of domain C is not well defined yet. Some researchers have suggested that it facilitates binding of the enzyme to the substrate and contributes to the interaction of the polysaccharide chain with the active site [19]. Apart from these three structural domains, some specialized α-amylases also exhibit different structural arrangements. For instance, the maltogenic amylase (MAA), belonging to the GH13_20 subfamily, features a unique structure consisting of five domains. Besides the A, B, and C domains mentioned above, MAA contains two additional domains which are responsible for binding to substrates. The maltose binding site of the domain E directs the polysaccharide chain to the active site and thereby promotes the degradation of starch to maltose [20,21,22].

Although there is low homology in the amino acid sequences of α-amylases from different sources, the highly conserved regions that compose the catalytic and major substrate binding sites are found in the primary structure of most of them. The active center of the enzyme consists of a binding center and a catalytic center, generally lying in the cavity between the domains A and B. The function of the binding site is substrate recognition and binding of the substrate, while the catalytic center is required for substrate hydrolysis and the release of the products. The catalytic center of α-amylase contains the highly conserved catalytic residues Asp and Glu which are present in almost every member of the GH13 family. Specifically, Glu acts as the general acid/base catalyst, while the Asp residues function as the catalytic nucleophile and transition-state stabilizer during the cleavage reactions [8]. This type of enzyme cleaves glycosidic bonds via a double-displacement mechanism, resulting in a net retention of the anomeric configuration of the sugar molecule; this is characteristic of “retaining” glycoside hydrolases [23]. Seminal biochemical analyses by Vallee et al. (1959) first established diverse α-amylases as calcium metalloenzymes, demonstrating that their native conformation and catalytic activity are strictly dependent on tightly bound calcium ions [24]. Under harsh industrial conditions such as starch liquefaction, the depletion of essential calcium ions is the direct structural determinant leading to the conformational collapse and rapid inactivation of the enzyme. The industrial application of α-amylase is partly limited by its strict structural dependence on calcium ions (Ca^2+^). In addition to the stabilizing role of Ca^2+^ as a key cofactor, protein-ligand interactions also play a vital role in maintaining its three-dimensional structural integrity [25]. The structure of the enzyme determines both its catalytic properties and its catalytic capacity; thus, it is essential to comprehend the relationship between the structure and function of α-amylases to guide their molecular modification and applications of α-amylases.

### 2.3. Kinetics and Mechanism of α-Amylase

The highly conserved architecture of the catalytic domain among α-amylases indicates that they share a common retaining mechanism for glycosidic bond cleavage. Specifically, GH13 α-amylases employ the classical Koshland double-displacement mechanism [23,26], resulting in a net retention of the anomeric configuration. Glycoside hydrolysis by this mechanism is achieved through two distinct steps involving an intermediate covalent link between the enzyme and the substrate (Figure 3b).

In the case of Taka-amylase A (TAA), during the initial glycosylation step, a glycosyl group in starch binds to the active site cleft. A glutamate (Glu230) in the catalytic triad acts as a general acid, donating a proton to the glycosidic oxygen during bond cleavage. Concurrently, an aspartate (Asp206) acts as a catalytic nucleophile, attacking the anomeric center to form a covalent glycosyl-enzyme ester intermediate via an oxocarbenium ion-like transition state. In the second (deglycosylation) step, an acceptor molecule (water or another carbohydrate) approaches the active center and attacks the covalent bond formed at the anomeric carbon of the −1 subsite. Thus, the ester bond is cleaved by water or glucose, with another aspartate (Asp297) acting as a transition state stabilizer by providing crucial electrostatic interactions [27,28]. When the acceptor molecule in this deglycosylation phase is a water molecule, hydrolysis occurs to release the products. Similarly, a transglycosylation reaction can take place if the acceptor molecule is a glycan molecule [29].

The retaining catalytic mechanism of GH13 α-amylases has been unequivocally demonstrated by numerous experiments [30]. These include covalent labeling using mechanism-based inhibitors like 4-deoxy-maltotriose-fluoride to capture the catalytic nucleophile, high-resolution X-ray crystallographic profiling of enzyme-substrate complexes, and site-directed mutagenesis of residues at the catalytic site [31,32].

## 3. Main Sources of α-Amylase

Amylases, the key enzymes catalyzing starch hydrolysis, are ubiquitously distributed across plants, animals, and microorganisms. In mammals, α-amylase acts as a primary digestive enzyme secreted by the salivary glands and pancreas to facilitate the breakdown of dietary starch [33]. In plants, α-amylases are prevalent within the Poaceae lineage (e.g., wheat, barley, and rice), where they play essential roles in seed germination and grain maturation [34].

For industrial applications, microorganisms serve as the predominant source of α-amylases owing to their rapid growth kinetics, cost-effective cultivation, and high amenability to genetic and metabolic engineering [35]. Bacteria and fungi constitute the primary microbial producers. Among bacteria, species of the Bacillus genus—including *Bacillus subtilis*, *Bacillus stearothermophilus*, *Bacillus licheniformis*, and *Bacillus amyloliquefaciens*—are highly efficient in synthesizing α-amylases with robust thermal stability and high specific activity, making them particularly suitable for high-temperature industrial processes [36]. Additionally, extremophilic bacteria, such as strains of *Bacillus stearothermophilus* and *Chromohalobacter* sp., secrete amylases that retain high activity under harsh conditions, thereby broadening their application potential in specialized industrial environments [37].

Furthermore, extremophilic α-amylases encompass cold-adapted variants of considerable biotechnological importance. A prominent example is the cold-active α-amylase (AHA) secreted by the Antarctic bacterium *Pseudoalteromonas haloplanktis*. Pioneering studies have extensively elucidated its structure–function relationships. They demonstrated that AHA achieves exceptionally high catalytic efficiency at low temperatures by evolving a highly flexible conformation characterized by reduced inter- and intramolecular interaction [38,39]. Moreover, this heat-labile enzyme exhibits a unique reversible two-state unfolding mechanism, wherein the active site constitutes its least stable region. From an industrial perspective, the superior low-temperature activity and facile thermal inactivation of such psychrophilic α-amylases make them highly valuable for energy-saving food processes, such as baking and brewing, where preventing undesirable post-processing over-saccharification is crucial.

Fungal-derived amylases are predominantly produced by filamentous fungi, notably *Aspergillus* and *Rhizopus* species. These fungi not only exhibit excellent enzyme-yielding capacities in solid-state fermentation (SSF) systems [40], but are also highly favored in the food industry due to their Generally Recognized As Safe (GRAS) status. For instance, *Aspergillus oryzae* and *Aspergillus niger* are widely exploited for the production of food-grade amylases and various other industrial enzyme preparations. Additionally, thermophilic fungi, such as *Thermomyces* species, have demonstrated significant potential in amylase production, yielding enzyme preparations with commendable thermal stability [41].

The commercial production of α-amylases predominantly employs two fermentation strategies: submerged fermentation (SmF), which is a conventional and well-established process, and solid-state fermentation (SSF), which represents a highly efficient emerging technological approach [42]. In the field of microbial α-amylases, extensive research has pioneered the optimization of SSF processes, demonstrating the immense potential of utilizing low-cost agro-industrial residues (e.g., wheat bran and spent brewing grains) as sustainable substrates. Furthermore, comprehensive empirical studies and reviews concerning their microbial sources, downstream processing, and industrial applications have established a critical baseline for modern enzyme biotechnology and large-scale commercialization [43,44,45].

## 4. Engineered α-Amylases with Altered Properties

The development of genetic engineering has enabled the precise modification of α-amylase’s properties, such as its stability and efficiency. This review systematically outlines the primary strategies for its molecular modification, including rational design and directed evolution, and discusses their specific aims in broadening the enzyme’s industrial applications.

### 4.1. Improving α-Amylase Yields

The widespread application of α-amylase across various sectors of the food industry has driven significant efforts to enhance its production yield through advanced enzyme engineering strategies. Recombinant DNA technology has played a crucial role in enabling the large-scale synthesis of food-grade α-amylases. By cloning and introducing highly efficient α-amylase genes into well-characterized and Generally Recognized as Safe (GRAS) microbial hosts such as *Bacillus subtilis* and *Pichia pastoris*, robust heterologous expression systems have been established. These systems not only significantly increase enzyme yields but also help improve functional properties critical for food applications, including enhanced thermal stability, optimal pH activity profiles, and reduced undesirable side activities. Consequently, recent research has focused on optimizing these expression platforms through multiple approaches, including using microbial strains with reduced protease production to minimize enzyme degradation, employing strong or food-grade inducible promoters for precise transcriptional control, and refining fermentation processes. These integrated genetic and bioprocess innovations have collectively contributed to the efficient and scalable production of high-performance α-amylases. Thus, heterologous expression in well-studied strains with reduced protease production, alongside promoter optimization and the use of inducible promoters, has been the focus of significant research efforts in recent years. Some amylase genes from different wild strains have been heterologously expressed in the widely used host *Escherichia coli*. The α-amylase of *Laceyella* sp. DS3 (AmyLa) exhibits moderate temperature stability, a wide pH range, and insensitivity towards Ca^2+^. Consequently, a recombinant AmyLa was produced in *Escherichia coli* BL21 (DE3). Heterologous expression of AmyLa in *E. coli* increases yield, and decreases fermentation costs by reducing the fermentation time, energy, and inducer concentration, while still facilitating its further molecular modification [46]. However, heterologous expression of proteins with *E. coli* as the host has certain drawbacks. For example, recombinant proteins occasionally accumulate in the cytoplasm as inclusion bodies [47]. Moreover, potential differences in codon preferences between *E. coli* and wild strains of α-amylase production may decrease expression efficiency and require codon optimization. The generation of endotoxin and acetate is also a disadvantage of *E. coli* as an expression host. Thus, *B. subtilis* is considered a high-quality host due to its non-pathogenicity and its ability to secrete recombinant proteins into the culture medium. Also, the majority of α-amylase producing bacteria belong to the genus *Bacillus*, therefore reducing codon usage issues. Therefore, it is believed that the *B. subtilis* expression system is more suitable for heterologous expression of α-amylase than *E. coli*. For instance, the α-amylase gene derived from *Geobacillus stearothermophilus* was heterologously expressed in *B. subtilis*. Through the synergistic optimization of key expression elements, the extracellular production in the engineered strain reached an exceptional 1244.17 U/mL, fully demonstrating the superiority of this host [48].

Fungal expression systems have some advantages such as high output at low cost, and they can install some post-translational modifications in the enzymes that bacteria are unable to perform. The *Pichia pastoris* expression system has been an important host for the production of secreted proteins in basic research and industrial processes [49]. Extracellular production of recombinant chimeric α-amylase (Ba-Gt-amy) was achieved in *P. pastoris* under the control of the AOX promoter. Ba-Gt-amy is a chimeric α-amylase constructed from the truncated acidic α-amylase of *Bacillus acidicola* (Ba-amy) and the thermostable α-amylase (Gt-amy) of *Geobacillus thermoleovorans* [50]. Ba-Gt-amy can undergo post-translational glycosylation for starch saccharification, releasing maltose, maltotriose, and other advanced maltooligosaccharides as major hydrolysis products [51].

In conclusion, cloning the natural gene encoding α-amylase and expressing it in an efficient microbial system can improve the production yields and it is a suitable platform that enables the molecular modification and design of recombinant proteins. For instance, the largest industrial enzyme manufacturer, the Danish company Novozyme (Bagsværd, Denmark), employs this strategy for α-amylase production [52]. The most relevant sources of heterologously expressed α-amylases are shown in (Table 1).

Sometimes, heterologous expression is not effective to boost α-amylases production and further molecular modification of the enzyme molecule is required. It is known that the secretion efficiency of α-amylases is influenced by the characteristics of the membrane phospholipid bilayer and the pIs of heterologous α-amylases, which affects the charge at a certain pH and therefore the electrostatic interactions that the enzyme establishes with other components. It is hypothesized that a better secretion yield can be obtained by reducing the electrostatic interactions between α-amylases and the cell surface. Following this strategy, upon inactivation of the phosphatidylserine synthase (PssA) and cardiolipin synthase (ClsA) at the cell surface, Cao et al. improved the secretion of α-amylases by up to 47% relative to the wild-type strain [66].

The availability of CRISPR technology has applications for the production of α-amylases through modifications at the genetic level in the producer strain. Geissler et al. applied the CRISPRi system to knock down 53 non-coding transcribed regions (ncRNA) that were differentially transcribed during the overexpression of α-amylase in *B. subtilis*. They found that two single guide RNAs (sgRNAs) targeted against a predicted antisense RNA of the 3′ UTR of the *skfA-skfH* operon, which mediates cell cannibalism upon nutrient limitation, increased amylase production by 21% [67]. Conversely, the sgRNAs targeting cytochrome c oxidase or sigma factor H mRNAs caused a severe reduction in the production of α-amylase. This illustrates that subtle changes in the production host, which are not often intuitive, can have a deep impact in the overall yield of heterologously produced proteins.

Furthermore, evaluating the true commercial utility of engineered α-amylases requires a shift beyond standard activity metrics towards comprehensive kinetic productivity analysis. Modifications that initially appear to lower intrinsic enzyme activity can actually double the overall reaction yield and long-term productivity due to enhanced sustained stability under industrial processing conditions [68].

### 4.2. Enhancing Catalytic Properties

#### 4.2.1. Modification by Molecular Techniques

Currently, protein engineering has become a tool for researchers who investigate the biological function of α-amylases and, simultaneously, allows their modification to improve their various catalytic properties. Enzyme engineering methods include non-rational modification, rational design, or semi-rational design, among others. Non-rational design adopts methods like random mutagenesis or genetic recombination to modify protein sequences and obtain mutant libraries. These techniques do not need extensive knowledge of the enzymes and are easily applied. After the library is created, a targeted screening is used to look for the desired enzymes, which concentrates most of the engineering efforts. Mutants BAA28 (D28E) and BAA294 (D28E/A294V) of *B. amyloliquefaciens* were constructed by the error-prone PCR technique. Their catalytic efficiencies (K_cat_/K_m_) were 43% and 61% higher than those of wild-type BAA, respectively, with 40% and 62% higher specific activities [69]. Cui et al. irradiated *B. subtilis* by heavy ion mutagenesis and obtained the mutant *B. subtilis* KC-180-2 after continuous cultivation. This strain produced an α-amylase activity of 72.26 U·mL^−1^, 82.34% higher than the original one [70].

Beyond single-round random mutagenesis, directed evolution serves as a highly effective strategy for α-amylase engineering. By mimicking natural evolutionary processes in vitro through iterative cycles of genetic diversification combined with high-throughput screening, researchers can achieve significant enzyme improvements without requiring prior structural knowledge. Advanced techniques such as DNA shuffling and the staggered extension process (StEP) are frequently utilized to this end. Specifically, DNA shuffling of homologous α-amylase genes has been instrumental in generating chimeric biocatalysts with broadened pH profiles, enhanced calcium independence, and superior thermostability, making them highly suitable for extreme industrial starch liquefaction. For instance, the gene reassembly of homologous wild-type sequences obtained via environmental screening yielded a high-performance chimeric α-amylase. This engineered enzyme operates optimally under harsh industrial conditions (pH 4.5 and temperatures up to 105 °C) without the requirement of exogenous calcium [71]. Consequently, the success of these directed evolution approaches relies on the continuous development of robust high-throughput screening assays, such as fluorescence-based microplate techniques or droplet-based microfluidics, to efficiently navigate the mutant sequence space and isolate variants with the desired industrial traits.

Rational design requires thorough comprehension of the α-amylase enzyme, including its function, primary and multiple-level structure, active center and catalytic mode, and the location of key amino acids. Rational engineering of *Bacillus amyloliquefaciens* substantially enhanced the production of acid-stable α-amylase. Employing a CRISPR-nCas9-mediated genome editing system, researchers eliminated autolysis-inducing prophage gene clusters and disrupted biosynthetic gene clusters for secondary metabolites that compete for precursor substrates, thereby augmenting cellular robustness and metabolic efficiency. In 5 L bioreactor fermentations, the engineered strain achieved a final α-amylase yield of 246,089.21 U/mL, representing a 2.78 times increase over the parental strain and demonstrating the efficacy of rational design in developing high-performance microbial cell factories [72]. For improving the catalytic properties and the acid-resistant capability of α-amylase of *B. licheniformis*, two key sites, Leu134 and Ser320, were identified by tridimensional structural analysis. Thus, the single mutants L134R and S320A, as well as the double mutant L134R/S320A were constructed. The enzymatic characterization revealed that L134R/S320A had the highest k_cat_/K_m_ at pH 4.5, which was approximately 14 times higher than that of the wild type. Meanwhile, the acid stability of L134R/S320A was significantly enhanced [73]. However, there are only a few examples of protein functional modifications relying solely on pure rational design due to the high expertise and technology requirements of this method.

Semi-rational design is based on a certain understanding of the physicochemical properties, tridimensional structure, and catalytic mechanism of proteins, and is based on fixed-point mutation, saturation mutagenesis, and combinatorial mutation of hotspot amino acids in the active pocket with the assistance of computers. Additional modifications in the expression host that enhance secretion and stability during translation and enzyme folding can also have a deep impact in this regard. The excellent enzymatic properties of α-amylase from *Bacillus stearothermophilus* (AmyS) have been investigated by many scholars. However, the heterologous extracellular production of AmyS in *B. subtilis* rendered a very low yield. Yao et al. designed a multistep approach to obtain improved AmyS. Thus, they screened for the best signal peptide for an α-amylase among 173 *B. subtilis* signal peptides, increasing the extracellular activity of the enzyme, with the accompanying production of a large number of inclusion bodies. They significantly reduced inclusion bodies formation and further increased AmyS activity by overexpressing intracellular molecular chaperones (GroEL–GroES and DnaK–DnaJ–GrpE). Finally, the mutant K82E/S405R (AmySA) was constructed by error-prone PCR. Implementation of the three strategies resulted in a 7.1 times improvement in AmyS activity compared to the initial strain [74]. Semi-rational design is an efficient protein modification strategy that involves identifying key targets based on structural information and then introducing local random mutations to those targets. At present, there are a large number of examples using this methodology to improve the enzymatic properties of α-amylases [75].

Although non-rational design circumvents the need for prior structural knowledge, it imposes prohibitive screening costs; conversely, purely rational design minimizes experimental efforts but heavily relies on rigorous structural elucidation, carrying a significant risk of functional disruption. In contrast, semi-rational design employs computational assistance to precisely construct focused ‘smart libraries,’ thereby effectively alleviating the screening burden while maximizing the success rate. Consequently, this synergistic approach, which optimally balances cost and efficacy, emerges as the most pragmatic and economically viable paradigm for enzyme engineering in current industrial applications.

#### 4.2.2. Modification by Physical and Chemical Methods

Beyond genetic engineering, the functional properties of α-amylases can be modulated through physical and chemical interventions. However, a rigorous distinction must be made between the use of non-covalent additives and true chemical modification. The application of stabilizers, such as metal ions and polyhydroxy compounds (PHCs), does not constitute chemical alteration; instead, these agents confer stability via non-covalent microenvironmental interactions. For instance, calcium, an essential cofactor for most α-amylases, stabilizes the enzyme conformation by promoting the salting-out of surrounding hydrophobic residues [76]. Consequently, optimizing calcium concentrations can significantly enhance enzymatic thermostability. As demonstrated by Pan et al., supplementing the amylase from *Bacillus stearothermophilus* (Bst-MFAse) with 10 mM Ca^2+^ and 40 mM Na^+^ preserved 71.1% of its residual activity following a 180 min incubation at 80 °C [77]. Beyond calcium and sodium, numerous studies suggest that the thermal stabilization of α-amylases by other metal ions is primarily achieved through the formation of electrostatic interactions with charged surface residues via specific and non-specific mechanisms [78]. Ions such as Mg^2+^, Co^2+^, Mn^2+^, Fe^2+^, Cu^2+^, Zn^2+^, and Ba^2+^ exert diverse effects on catalytic activity and stability. For example, Bharwad et al. investigated an α-amylase from a hot-spring metagenome, revealing that low concentrations of Mg^2+^ and Co^2+^ promoted hydrolysis, whereas Cu^2+^, Mn^2+^, Fe^2+^, Hg^2+^, and Ni^2+^ acted as inhibitors [79]. Furthermore, the modulatory effect of metal ions is highly concentration- and source-dependent. Excessive metal concentrations often induce toxicity, resulting in reduced activity or complete inactivation. Similarly, identical ions can yield contrasting effects across different microbial sources; for instance, 1 mM Co^2+^, Ba^2+^, and Ag^+^ severely inhibited the α-amylase activity of *Streptomyces gulbargensis* DAS 131 by nearly 70% [80]. Thus, the application of non-covalent metal stabilizers requires individualized empirical optimization based on the specific enzyme type and operational mode.

Conversely, true chemical alteration entails the direct covalent modification of specific amino acid residues to reinforce structural rigidity. This strategy is particularly advantageous for stabilizing inherently heat-labile biocatalysts. Previous seminal studies on the cold-adapted α-amylase (AHA) from the Antarctic bacterium *Pseudoalteromonas haloplanktis* perfectly exemplify this approach. By employing targeted covalent modifications—such as the specific derivatization of surface residues and the introduction of chemical cross-links—they successfully restricted the extreme conformational flexibility of the psychrophilic enzyme [81]. These covalent interventions significantly augmented the enzyme’s thermodynamic and kinetic stability, thereby maximizing its catalytic efficiency and operational productivity. Ultimately, targeted chemical modification serves as a potent and complementary alternative to genetic mutagenesis for the rational design of robust industrial enzymes.

Physical alterations of α-amylase are mainly carried out by ultrasound, irradiation, high pressure, and pulsed electric fields, which have a positive or negative effect on α-amylase properties by changing the structure of the enzyme or altering non-covalent bonds in the molecule. Abedi et al. significantly improved the catalytic properties of the α-amylase from *B. licheniformis* using Ca^2+^ (25 mM) and ultrasound (64.5 W, 25 + 40 kHz). In these conditions, the half-life of α-amylase was 113.7% compared to the control. Ultrasound-treated α-amylase showed a 14% increase in α-helices and a 39% decrease in β-sheet structures compared to the untreated enzyme [82]. Rather than through direct photon absorption, low-level laser irradiation (LLLI) indirectly modulates α-amylases activity via microenvironmental alterations, yielding a wavelength- and dose-dependent bidirectional effect that enhances activity at 589 nm (20 J/cm^2^) while suppressing it at 532 nm (40 J/cm^2^) [83]. Some studies revealed that microwave irradiation may lead to a decrease in α-amylase’s α-helical content and an increase in β-turn, β-sheet, and random coil contents, which contribute to alterations in the forces that stabilize the enzyme (hydrogen bonds, van der Waals forces, and hydrophobic interactions), thereby affecting the enzyme’s thermostability and other properties [84]. Gamma ray-mediated mutagenesis, which replaced leucine 203 with isoleucine at the active site of glucoamylase from *Aspergillus oryzae*, significantly enhanced the enzyme’s thermostability. The mutant M-60(5) exhibited a 1.92 times extension in half-life at 55 °C and an increase in the free energy of thermal denaturation by 3.43 kJ mol^−1^, indicating that the mutation induced conformational changes in the active site, thereby reinforcing structural rigidity and reducing thermal inactivation at elevated temperatures [85]. Pulse electric field (PEF), like irradiation, affects enzyme properties primarily by altering the secondary, tertiary, and quaternary structure of the enzyme. High pressure has a significant effect on amylase stability and activity, but the mechanisms are not fully understood. One possible explanation is that high pressure increases the density of the first hydration shell on the protein surface and restricts lateral chain movement [86]. While physical methods offer promising avenues for modulating α-amylase function, their efficacy is often constrained by the necessity for precise control over critical parameters. Inappropriate application can induce detrimental effects, such as structural denaturation or functional impairment of the enzyme. Consequently, extensive optimization is imperative to identify conditions that maximize beneficial outcomes while preserving enzymatic stability and activity.

#### 4.2.3. Enzyme Immobilization for Industrial Applications

Enzyme immobilization enhances the utilization of biocatalysts through continuous and repeated operational cycles by confining enzymes to a specific phase or solid carrier. The industrial application of free α-amylases is frequently impeded by their inherent instability and single-use limitations under harsh processing conditions, such as extreme pH and high temperatures. Immobilization presents a robust strategy to circumvent these hurdles. Anchoring the enzyme onto a solid matrix restricts its conformational flexibility, thereby augmenting structural rigidity and resistance to thermal or chemical denaturation. Furthermore, this technique facilitates facile downstream recovery and recycling of the biocatalyst, which significantly reduces operational costs and enables continuous manufacturing processes [87]. Consequently, the development of cost-effective, easily controllable, and highly stable immobilized α-amylases has emerged as a major research hotspot in the food, pharmaceutical, and chemical industries.

Typical immobilization methods include covalent attachment, embedding, adsorption, covalent binding, and cross-linking [88]. Covalent adsorption can lead to increased structural rigidity of the enzyme and prevent enzyme autolysis by immobilizing the enzyme space, which is also relatively effective for enhancing thermal stability [89,90]. Ashly et al. studied the immobilization of α-amylase on polyaniline (PANI) by covalent binding and physical adsorption. It was demonstrated that the immobilized enzyme retained nearly 90% activity after 120 min at the optimum temperature, whereas the free enzyme conserved less than 40% activity. In addition, the catalytic efficiency and storage stability of the immobilized enzyme were also significantly improved [91]. Recently, Razzaghi et al. covalently immobilized the α-amylase from *Bacillus aquimaris* on synthesized cellulose/gold hybrid nanoparticles (CNC/Au) and determined its stability and catalytic efficiency. Compared to the free enzyme, the optimum temperature of the immobilized enzyme was increased by 10 °C, and its residual activity was increased by 17%, 14%, and 17% at 70, 80, and 90 °C, respectively. Moreover, the catalytic efficiency of the immobilized enzyme was also improved. The enhanced rigidity is largely attributed to the robust covalent linkages formed between the functionalized supports and the surface amino acid residues of the enzyme [92]. Beyond organic polymers, carbon-based and inorganic nanomaterials have demonstrated exceptional potential. Covalently conjugating a calcium-independent α-amylase onto genipin-modified multi-walled carbon nanotubes and silica supports extended the enzyme’s half-life by 4.1-fold and maintained over 85% residual activity after 10 continuous cycles [93]. Similarly, the nanoconfinement of α-amylase within mesoporous silica [94] or bentonite matrices [95] has proven highly effective in preserving the enzyme’s native fold, leading to a three-fold increase in half-life and sustained catalytic activity toward size-matched substrates. Metal–organic frameworks (MOFs), such as ZIF-8 nanocomposites, have also been successfully employed, enabling the enzyme to maintain over 60% activity at 80 °C and exhibit superior tolerance to extreme pH conditions [96].

To further optimize industrial applicability, magnetic nanoparticles (MNPs)—particularly Fe_3_O_4_-based nanocomposites—have emerged as highly efficient immobilization carriers. The defining advantage of MNPs is their outstanding recyclability; the biocatalyst can be rapidly and effortlessly separated from the reaction mixture via an external magnetic field, circumventing the need for costly and time-consuming centrifugation or filtration [97]. Additionally, the large surface-to-volume ratio and tunable surface functionalization of MNPs facilitate high enzyme loading capacities and robust covalent binding [98]. Recently, an integrated approach combining thermally adapted extremophilic enzymes, targeted genetic modifications, and MNP immobilization has been proposed as a unified strategy to synergistically maximize biocatalytic stability and cost-effectiveness [99].

While enzyme immobilization on advanced matrices—such as organic polymers, mesoporous nanomaterials, and magnetic nanocomposites—significantly augments the structural rigidity and recyclability of α-amylase, its commercial translation remains critically bottlenecked. To bridge the gap between idealized laboratory models and continuous industrial manufacturing, future research must rigorously address severe mass-transfer limitations in highly viscous substrates, prohibitive scale-up costs, and stringent food safety regulations regarding nanomaterial toxicity.

**Figure 4:**
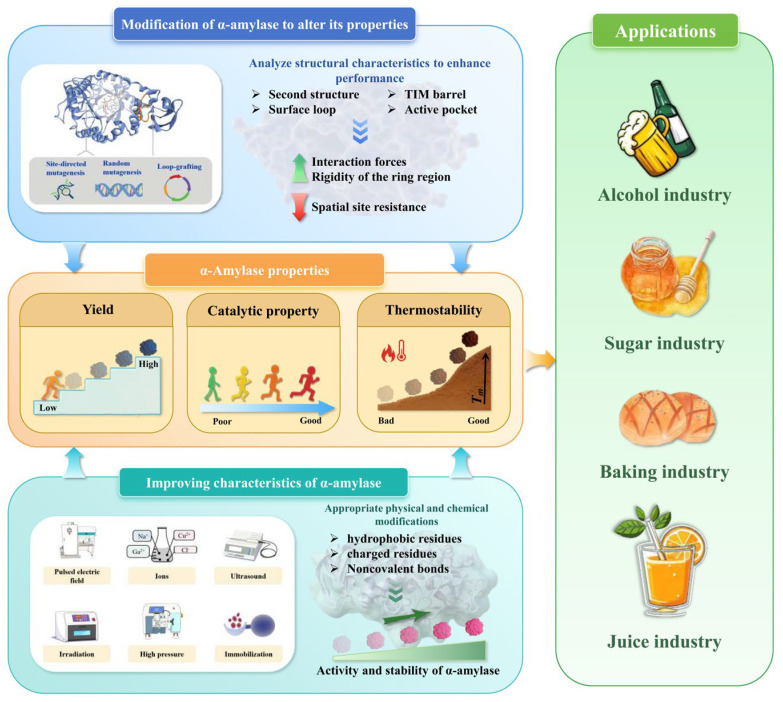
Modification of α-amylase and its application in food industry.

In summary, the enhancement of α-amylase thermostability via immobilization is fundamentally driven by the rigidification of the enzyme structure upon binding to a carrier surface, which minimizes detrimental conformational unfolding under extreme conditions and mitigates autolysis (Figure 4).

### 4.3. Improving the Thermal Stability

The thermal stability of α-amylases is highly relevant in the washing, food, textile, paper, and feed industries, which frequently require elevated operating temperatures to accelerate reaction rates, reduce fluid viscosity, and prevent microbial contamination [29]. While microbial α-amylases inherently possess greater stability than their plant and animal counterparts [100], meeting stringent industrial demands necessitates further engineering of their three-dimensional structures.

However, a fundamental bottleneck in engineering thermostability is the pervasive “activity-stability trade-off.” Enhancing thermal resistance typically requires augmenting structural rigidity, which can simultaneously compromise the conformational flexibility necessary for efficient catalysis. Therefore, successful engineering must carefully target specific regions. For instance, modifying amino acids near the active pocket can strengthen local interactions and reduce steric hindrance without destroying global flexibility. This was demonstrated by constructing a mutant (MuBAA) that mitigated calcium depletion-induced conformational collapse, successfully balancing augmented structural rigidity with a 99.1% improvement in specific activity [101]. Similarly, *Rhizopus oryzae* α-amylase (ROAmy) was aligned with other thermostable or acid-resistant fungal α-amylases displaying high sequence similarity via a multiple sequence alignment (MSA)-based fixed-point evolution approach. By comparing specific amino acid residues—especially near the active site—corresponding mutation sites were designed. Four mutants (A144Y, V174R, T253E, and I276P) were screened for thermal stability, acid resistance, and catalytic efficiency. Ala144 is a hydrophobic amino acid located on the surface of the irregularly curled Domain B, which is substituted by the polar residue Tyr to reduce the hydrophobicity of the surface of the protein. This correlates with an optimal temperature increase from 50 to 55 °C and the optimal pH changing from 5.5 to 5.0. The mutants V174R and T253E formed additional hydrogen bonds and salt bridges with surrounding residues, resulting in 2.52- and 1.65-fold increases of the half-life (t_1/2_) at 55 °C, respectively. In the mutant I276P, although there is a decrease of hydrogen bonds, the introduction of the structure-disrupting residue Pro may enhance the rigidity of the irregularly curled region of the structural domain A, which leads to 1.35-fold increases of the t_1/2_ compared with the wild-type [102]. Furthermore, systematic engineering of multiple highly flexible regions (HFRs)—such as N-terminal truncation combined with saturation mutagenesis—has proven to be a highly effective strategy to precisely balance enzymatic thermostability and catalytic efficiency [103]. Ultimately, increased intramolecular interactions—such as enhanced hydrophilicity, additional hydrogen bonds, salt bridge formation, and the introduction of rigidity in flexible regions—are predominantly responsible for the elevated thermal stability of α-amylases.

α-amylases are typical TIM barrel (β/α)8-barrel) enzymes. The catalytic and substrate-binding residues of the TIM barrel constitute the “catalytic face,” which includes the C-terminal ends of the β-strands and their extending loops (Figure 3a). Conversely, the stabilizing residues are located on the “stability face,” encompassing the hydrophobic core, the N-terminal ends of the barrel, and the αβ-loops linking the α-helices with the subsequent β-strands [104]. Beneficial mutations contributing to the thermal stability and catalytic activity of α-amylases are primarily distributed across these two faces. To obtain an α-amylase BLA from *B. licheniformis* WX-02 with superior thermal stability and activity, Cui et al. rationally designed the Q360C mutant. This variant exhibited a residual activity of 75% after preincubation at 70 °C for 30 min, outperforming the wild-type (59%). This thermostability improvement is likely explained by a significant enhancement of hydrophobic interactions near the mutation site following the substitution of Gln360 with Cys [105]. Enhancing interactions between catalytic and stabilizing residues in the barrel core contributes to the overall formation and stabilization of the TIM barrel, thereby improving the enzyme’s thermostability.

Critically, the central eight β-strands constituting the TIM barrel core form a highly conserved structure that plays a crucial role in enzyme stability. Traditionally, this region has been considered “off-limits” and is rarely targeted for protein engineering due to the high risk that any structural perturbation might lead to a catastrophic loss of protein functionality and catalytic geometry. Challenging this paradigm, Wang et al. designed a mutation (V260I) at the β7-strand, which successfully increased thermal stability by 7.1 °C compared to the wild-type α-amylase. This enhanced stability is driven by broader weak interactions between the central β-strands, including hydrogen bonds, C-H…O, C-H…π, O-H…π, and N-H…π interactions [106]. Such augmented rigidity within the core region directly counteracts the thermal denaturation stress induced by prolonged high temperatures during industrial saccharification. Therefore, engineering the central eight β-sheets of the TIM barrel to optimize specific tertiary interactions serves as a viable and novel strategy to enhance the enzymatic properties of α-amylases, an approach that could be broadly applied to other TIM barrel proteins.

Traditional evolutionary approaches for improving the thermal stability of α-amylases, such as random single or multiple point mutations, have typically provided limited, incremental improvements. Today, computer-aided design enables structural rearrangements via the loop-grafting of amino acids, which repairs structurally defective flexible regions to generate heat-resistant enzymes. Zhu et al. screened the α-amylase of *Geobacillus stearothermophilus* DSMZ 456 using molecular dynamics (MD) simulations and replaced structurally defective regions with corresponding segments from homologous thermophilic α-amylases featuring optimal temperatures above 85 °C. Following the screening of promising mutants via thermal unfolding and MD simulations, the LOOP (loop2-1bli) mutant—constructed by replacing the wild-type loop 2 fragment (K298-Q317) with the homologous fragment (A298-L317) from 1bli—exhibited remarkable thermal stability, extending its half-life 8-fold at 100 °C [107]. Despite its superior efficacy in achieving drastic structural leaps, this loop-grafting strategy is heavily constrained by the availability of high-resolution homologous templates and demands intensive MD simulations to predict grafting compatibility. By replacing small portions of flexible regions, this loop-grafting strategy proves to be more powerful and effective than conventional point mutations, and it can be simultaneously applied to improve other properties such as catalytic efficiency, enzyme-substrate affinity, and specific activity (Table 2).

## 5. Applications, Challenges, and Industrial Perspectives of α-Amylase in the Food Sector

In the food industry, α-amylase occupies a critical position by being instrumental in the efficient utilization of starch. It performs an essential role in a wide array of food processing sectors such as baking, sugar refining, alcoholic fermentation, and juice extraction. The applications of α-amylases in these industries lead to better product quality and reduce the use of chemicals, which protect human health and meet the consumer requirements for less additives [100]. A detailed overview of the use of α-amylases in different food fields is presented below (Figure 4).

### 5.1. Application of α-Amylase in Bread Improvement

α-Amylase, as a modifier, is used in bread processing to improve the fermentation rate, increase the volume of bread, reduce the hardness of the core and make the core soft and elastic, and change the color and flavor of the bread, which collectively contribute to the quality of bread [114]. During the dough fermentation process, an appropriate α-amylase is added to hydrolyze the starch in the dough to dextrin, which is then degraded by yeasts. The properties of the starch in the bread dough are changed, and the high-strength gluten structure is destroyed. This causes the elongation of the walls of the small air chambers in the dough which, in turn, increases the volume of the bread during baking. Enzymatic digestion of high molecular weight starch also improves the fermentation efficiency and reduces the viscosity of bread doughs during manufacturing [115]. The small molecule dextrin produced by the degradation of starch by α-amylase can interfere with the interaction between starch and gluten, which makes the texture of bread softer and prolongs the shelf life of bread [114]. Meanwhile, the sugars formed by α-amylases hydrolysis of starch, such as maltose and glucose, can take part in the Maillard reaction and caramelization during the baking process to improve the flavor and color of bread [116].

Currently, α-amylases that produce malt oligosaccharides have attracted great attention for their applications in bread baking. Malt oligosaccharides not only have good adaptability to food processing but also have a variety of functions that are beneficial to human health. Bae et al. utilized an α-amylase that produces maltotetraose (G4-amylase) to improve the physicochemical properties of whole-wheat bread. The study showed that a variety of malt oligosaccharides were produced in bread with G4-amylase compared to the control, in which maltotetraose predominated. The anti-aging properties of the bread are also improved owing to the presence of malt oligosaccharides [117]. AmyM-TR2, a maltohexaose-producing amylase obtained by truncation of the CBM20 structural domain of the α-amylase from *Corallococcus* sp. strain EGB, was applied to bread production. The bread dough treated with AmyM-TR2 showed better physicochemical properties such as a larger volume, better texture, and longer shelf life [118]. However, the addition of α-amylases should be controlled or, otherwise, excessive branched dextrins will be produced, which will cause quality problems in the bread center [119].

### 5.2. Application of α-Amylase in Sugar Industry

Most industrial starch-to-sugar production is a sequential enzymatic process consisting of three key stages: high-temperature dextrinization (105 °C for 5 min) to gelatinize and rupture starch granules; liquefaction (95 °C for 2 h) where α-amylase breaks down long starch chains into shorter dextrins to significantly reduce viscosity; and finally, a prolonged saccharification phase (55–65 °C for 48–72 h), wherein glucoamylase completely hydrolyzes the dextrins into simple sugars [120]. Starch-based sugar products mainly include glucose, maltose syrup (caramel, high maltose syrup, maltose), maltodextrin, malt oligosaccharides, and fructose syrup, which require the participation of α-amylases along with other types of hydrolases. Glucose is produced mainly by the combined action of α-amylase, glucoamylase, and pullulanase. Maltose is mainly produced by using α-amylases and β-amylases. β-amylase acts on the α-1,4-glycosidic bond at the non-reducing terminus of the starch or dextrins to sequentially cut down the maltose as well as converting the generated disaccharide from the α- to the β-configuration. So far, some novel α-amylases have been discovered that hydrolyze malt oligosaccharides directly from starch, such as the maltohexaose-producing amylase AmyM [118]. Glucose isomerase plays a key role in the preparation of fructose syrup by isomerizing glucose to fructose. Researchers treated purple sweet potato starch with α-amylase, xylanase, and amyloglucosidase (AMG) and produced the maximum total reducing sugar concentration of 143.98 and 68.91 g/L after 48 h [121]. Lee et al. used a mix of starch-degrading enzymes, including α-amylase, and fungal-degrading enzymes to decompose food waste infected with fungi. The research suggested that this method is not only effective for degrading food waste, but it was also able to directly render 62.44 mM glucose. Thus, a new method of producing sugar from waste in the industrial sector has been developed [122]. Fungal α-amylases are widely used in the sugar industry due to their excellent hydrolytic properties. However, they are generally poorly thermostable, which increases the additional production cost due to loss of enzyme activity during production process.

### 5.3. Application of α-Amylase in Alcohol Industries

Baijiu fermentation requires the microbiota provided by the Daqu, which secretes various hydrolytic enzymes to saccharify the grain material. The saccharification is the first step in the production of Baijiu [123]. Xia et al. investigated the main enzymes involved in the glycation process by metaproteomics. It was found that the amylase system (α-amylase, α-glucosidase, and glucoamylase) and the cellulase system (cellobiohydrolases, β-glucosidase, and endoglucanase) played important roles in this stage [124]. The flavor compounds in Baijiu are mainly esters, alcohols, phenols, aldehydes, and organic acids, among other aromatic compounds [125]. The higher alcohols in Baijiu are monohydric alcohols containing more than three carbons. It can provide Baijiu with a special aroma and play a role in setting off the ester flavor. However, excessive content of higher alcohols can lead to spicy bitterness and endanger human health [126]. Researchers found that α-amylase can effectively regulate the content of higher alcohols. Luo et al. investigated the effect of α-amylase addition on the production of higher alcohols. After adding a series of concentration gradients of α-amylase into the fermentation tank for 32 days, it was detected that the yield of Baijiu increased while the production of higher alcohols remained unchanged. During Baijiu fermentation, the addition of a certain percentage of α-amylase, saccharase, and yeast reduced the content of higher alcohols by 21.06% compared to the control. In conclusion, α-amylase can not only improve the yield of liquors by hydrolyzing starchy raw materials but also improve the quality of Baijiu by reducing the content of higher alcohols.

In brewing beer, α-amylase can promote the hydrolysis of starch and improve the quality of the beer as an enzymatic agent. The temperature of the saccharification process is generally increased from 55 to 70 °C, and the yield of fermentable sugars is maximized after about 20 min. By accurately adjusting the saccharification time and the corresponding temperature at the raw material treatment stage, it is possible to shorten the fermentation time, and optimally improve the activity of hydrolytic enzymes like α-amylase and β-amylase [127].

### 5.4. Critical Assessment and Industrial Challenges of α-Amylase Applications

Despite its extensive utility, the industrial application of α-amylase necessitates a critical evaluation of enzyme sourcing, dosage limits, and economic feasibility. In baking, precise control of α-amylase addition is crucial; excessive activity leads to the overaccumulation of branched limit dextrins, which causes severe quality defects in the bread center, such as a gummy crumb structure [128]. Furthermore, enzyme efficacy must be rigorously matched to specific process conditions. For instance, while fungal α-amylases exhibit excellent hydrolytic properties, they are generally poorly thermostable, which increases production costs due to activity loss in high-temperature processes like sugar liquefaction [129]. Conversely, highly thermostable bacterial α-amylases are ideal for high-heat environments but pose significant risks of over-hydrolysis if used indiscriminately in baking. Beyond efficacy, the transition of engineered α-amylases from laboratory to industrial scale is impeded by the high costs of downstream purification and the mass-transfer limitations of immobilization carriers in large bioreactors. Finally, stringent regulatory frameworks demand that food-grade enzymes be derived from Generally Recognized as Safe (GRAS) organisms [130], posing a substantial compliance hurdle for high-yield recombinant strains that may harbor residual genetic material [131].

## 6. Conclusions and Future Research

As a pivotal industrial biocatalyst, the applications of α-amylase have significantly expanded through profound structural elucidation and targeted molecular modifications. Recent advances have objectively demonstrated that integrating semi-rational design, structural flexibility engineering, and novel immobilization techniques can successfully overcome critical operational bottlenecks. These multi-dimensional strategies have markedly enhanced the enzyme’s thermal stability, catalytic efficiency, and reusability, thereby streamlining modern food processing. To balance these established achievements with future industrial demands, subsequent research must prioritize artificial intelligence-driven molecular engineering to confer extreme environmental robustness and precise product specificity. Ultimately, overcoming the economic challenges of downstream purification and ensuring the rigorous GRAS compliance of engineered strains will be essential for fully leveraging the biocatalytic potential of α-amylases in sustainable industrial applications.

## Figures and Tables

**Table 1:** Heterologous expression of α-amylases from different sources.

Wild Type Strain	Recombinant Strain	Optimum Temperature (°C)	Optimum pH	Yield	References
*Laceyella* sp. DS3	*E. coli* BL21 (DE3)	55	6.0	41.83 U/mg	[46]
*Bacillus acidicola*	*Pichia pastoris* X-33	60	4.0	750 ± 4.5 U/mL	[51]
*Geobacillus thermodenitrificans* DSM-465	*E. coli* BL21 (DE3)	70	8.0	153.6 U/mg	[53]
*Paenibacillus* sp. D9	*E. coli BL21* (DE3)	30	7.0	451 U/mg	[54]
*Anoxybacillus vranjensis* ST4	*E. coli* ER2523	60–80	4.5–8.5	336.8 IU/mg	[55]
*Bacillus amyloliquefacien* JH-06	*B.subtilis* WB800	60	6.0	5566 U/mg	[56]
*Bacillus licheniformis* 104.K	*E. coli Rosetta* (DE3)	90	6.0	163 U/mL	[57]
*Bacillus stearothermophilus*	*B.subtilis* WB800	65	5.5	198.73 U/mL	[58]
*Staphylothermus marinus*	*B. subtilis* ISW1214	/	/	66.96 U/mL	[59]
*Pyrococcus furiosus*	*B. subtilis* WS9	/	/	3806.7 U/mL	[60]
*/*	*B. subtilis* 168			591.4 U/mL	[61]
alkaliphilic Alkalimonas *amylolytica* N10	*Pichia pastoris* GS115	55	9.5	16.6 U/mg	[62]
*Corallococcus* sp. strain EGB	*Pichia pastoris* GS115	/	/	220 mg/L	[63]
*Aspergillus oryzae strain* S2	*Pichia pastoris* SMD1168H	60	5.6	72 U/mL	[64]
*Penicillium brevis NRRL864*	*Escherichia coli*	55	6.0	195 U/mL	[65]

**Table 2:** Results of host manipulation and protein engineering on α-amylase.

Microorganism/α-Amylase	Approaches/Alterations	Significant Improvement	References
Improving α-amylase yields			
*Pyrococcus furiosus*	Chaperone Co-Expression by CRISPR/Cas9	A 1.2-fold increase in the extracellular yield of α-amylase	[60]
*B. subtilis* 168	ARTP mutagenesis and high throughput screening	α-Amylase yield was increased from 1.31 U/(mg·h) to 1.57 U/(mg·h)	[61]
*B. subtilis* DB104/amyE	Cell surface engineering	The secretion of *amyE* increased by 47%	[66]
*Bacillus halmapalus*/JE1	CRISPRi screen	A 21% increase in α-amylase JE1 yield	[67]
*B. subtilis*	Knockout of the *flgE* gene by CRISPR-dCas9	Approximately 30% increase in α-amylase production	[108]
*B. subtilis WB600*/AmyZ1	Modification of expression regulatory elements	A 2.5-fold increase in α-amylase yield	[109]
*B. amyloliquefaciens* TCCC11018/BLA	Knockout of related peptidoglycan hydrolase genes	The mutant BA Δ*SDE* yield of 7851.53 U/mL was significantly higher than wild type	[110]
Enhancing catalytic property			
*Bacillus amyloliquefaciens*/BAA	error-prone PCR	K_cat_/K_m_ 43 and 61% higher than wild-type BAA	[69]
*Bacillus subtilis* 20076/ KC-180-2	heavy ion mutagenesis	The KC-180-2 activity was enhanced by 82.34%	[70]
*Bacillus licheniformis*/BLA	L134R/S320A	The k_cat_/K_m_ of the mutant BLA is about 14 times larger than that of the wild-type	[73]
*Bacillus stearothermophilus*/AmyS	Signal peptide screening, molecular chaperone overexpression, and error-prone PCR	The AmyS activity was improved by 7.1-fold	[74]
*Bacillus siamensis* JJC33M/AmyJ33	truncation mutation	Deletion of structural domains D and E results in a 4.9-fold increase in K_cat_/K_m_ of AmyJ33	[111]
Improving the thermal stability			
*Bacillus amyloliquefaciens*/BAA	Multiple fixed-point mutagenesis	BAA activity was 99.1% higher than wild type at 50 and 60 °C	[101]
*Rhizopus oryzae*/ROAmy	Multiple fixed-point mutagenesis	1.35-fold increase in t_1/2_ of ROAmy	[102].
*Bacillus licheniformis* WX-02/BLA	Q360C by rational design	The residual activity of the mutant incubated at 70 °C for 30 min was 16% higher than that of the wild-type	[105]
*Bacillus subtilis* CN7/Amy7C	Altering amino acids on (β/α)_8_ barrels	7.1 °C improved the thermal stability of mutant V260I	[106]
*Geobacillus stearothermophilus* DSMZ 456	Loop-grafting	8-fold increase in t_1/2_	[107]
*Alkalimonas amylolytica* N10/AmyK	Addition of disulfide bonds	A 6-fold increase in t_1/2_ and 5.2 °C increase in Tm of the mutant	[112]
*Pseudomonas saccharophila STB07*/MFAses	Addition of disulfide bonds	A 2.6-fold increase in t_1/2_ at 60 °C	[113]

## Data Availability

No new data were created or analyzed in this study. Data sharing is not applicable to this article.

## References

[1] van der Maarel M.J.E.C., van der Veen B., Uitdehaag J.C.M., Leemhuis H., Dijkhuizen L. (2002). Properties and Applications of Starch-Converting Enzymes of the α-Amylase Family. J. Biotechnol..

[2] Drula E., Garron M.-L., Dogan S., Lombard V., Henrissat B., Terrapon N. (2022). The Carbohydrate-Active Enzyme Database: Functions and Literature. Nucleic Acids Res..

[3] Mikami B., Hehre E.J., Sato M., Katsube Y., Hirose M., Morita Y., Sacchettini J.C. (1993). The 2.0-.ANG. Resolution Structure of Soybean .Beta.-Amylase Complexed with .Alpha.-Cyclodextrin. Biochemistry.

[4] Roth C., Moroz O.V., Ariza A., Skov L.K., Ayabe K., Davies G.J., Wilson K.S. (2018). Structural Insight into Industrially Relevant Glucoamylases: Flexible Positions of Starch-Binding Domains. Acta Crystallogr. D Struct. Biol..

[5] Miłek J., Lamkiewicz J. (2022). The Starch Hydrolysis by α-Amylase Bacillus Spp.: An Estimation of the Optimum Temperatures, the Activation and Deactivation Energies. J. Therm. Anal. Calorim..

[6] Tesfaye E.L., Kumar P., Jutur P.P., Tefera A.T., Jiru T.M., Gaur N.A. (2025). The Role of Amylase in Bioethanol Production: Advances in Amylase-Producing Strains Using CRISPR/Cas9 Technology. Fuel Commun..

[7] Stam M.R., Danchin E.G.J., Rancurel C., Coutinho P.M., Henrissat B. (2006). Dividing the Large Glycoside Hydrolase Family 13 into Subfamilies: Towards Improved Functional Annotations of α-Amylase-Related Proteins. Protein Eng. Des. Sel..

[8] Janeček Š., Svensson B., MacGregor E.A. (2014). α-Amylase: An Enzyme Specificity Found in Various Families of Glycoside Hydrolases. Cell. Mol. Life Sci..

[9] Poláček A., Lombard V., Coutinho P.M., Terrapon N., Janeček Š. (2025). Dividing the α-Amylase Family GH57 of Starch Hydrolases and Related Enzymes into Subfamilies Using Evolutionary, Clustering and Functional Criteria. Int. J. Biol. Macromol..

[10] Lombard V., Golaconda Ramulu H., Drula E., Coutinho P.M., Henrissat B. (2014). The Carbohydrate-Active Enzymes Database (CAZy) in 2013. Nucleic Acids Res..

[11] Baroroh U., Yusuf M., Rachman S.D., Ishmayana S., Hasan K., Subroto T. (2019). Molecular Dynamics Study to Improve the Substrate Adsorption of *Saccharomycopsis fibuligera* R64 Alpha-Amylase by Designing a New Surface Binding Site. AABC.

[12] Janeček Š., Mareček F., MacGregor E.A., Svensson B. (2019). Starch-Binding Domains as CBM Families–History, Occurrence, Structure, Function and Evolution. Biotechnol. Adv..

[13] Cockburn D.W., Suh C., Medina K.P., Duvall R.M., Wawrzak Z., Henrissat B., Koropatkin N.M. (2018). Novel Carbohydrate Binding Modules in the Surface Anchored α-Amylase of *Eubacterium rectale* Provide a Molecular Rationale for the Range of Starches Used by This Organism in the Human Gut. Mol. Microbiol..

[14] Zeng J., Guo J., Tu Y., Yuan L. (2020). Functional Study of C-Terminal Domain of the Thermoacidophilic Raw Starch-Hydrolyzing α-Amylase Gt-Amy. Food Sci. Biotechnol..

[15] Valk V., van der Kaaij R.M., Dijkhuizen L. (2017). The Evolutionary Origin and Possible Functional Roles of FNIII Domains in Two *Microbacterium Aurum* B8.A Granular Starch Degrading Enzymes, and in Other Carbohydrate Acting Enzymes. Amylase.

[16] Vuillemin M., Moreno Prieto E.S., Pilgaard B., Siebenhaar S., Holck J., Henrissat B., Bahieldin A., Hakeem K.R., Alghamdi K.M. (2024). Biochemical Exploration of Family GH119 Reveals a Single α-Amylase Specificity and Confirms Shared Catalytic Machinery with GH57 Enzymes. Int. J. Biol. Macromol..

[17] Hodorová M., Janeček Š. (2024). The Family GH126–Its Relatedness to and Differentiation from GH8 and GH48 Including the Intermediary Sequences. Food Biosci..

[18] Matsuura Y., Kusunoki M., Harada W., Kakudo M. (1984). Structure and Possible Catalytic Residues of Taka-Amylase A. J. Biochem..

[19] Holm L., Koivula A.K., Lehtovaara P.M., Hemminki A., Knowles J.K.C. (1990). Random Mutagenesis Used to Probe the Structure and Function of *Bacillus stearothermophilus* Alpha-Amylase. Protein Eng. Des. Sel..

[20] Christiansen C., Abou Hachem M., Janeček Š., Viksø-Nielsen A., Blennow A., Svensson B. (2009). The Carbohydrate-binding Module Family 20–Diversity, Structure, and Function. FEBS J..

[21] Han R., Li J., Shin H.-D., Chen R.R., Du G., Liu L., Chen J. (2013). Carbohydrate-Binding Module–Cyclodextrin Glycosyltransferase Fusion Enables Efficient Synthesis of 2-*O*-D-Glucopyranosyl-L-Ascorbic Acid with Soluble Starch as the Glycosyl Donor. Appl. Environ. Microbiol..

[22] Penninga D., Van Der Veen B.A., Knegtel R.M.A., Van Hijum S.A.F.T., Rozeboom H.J., Kalk K.H., Dijkstra B.W., Dijkhuizen L. (1996). The Raw Starch Binding Domain of Cyclodextrin Glycosyltransferase from *Bacillus circulans* Strain 251. J. Biol. Chem..

[23] Koshland D.E. (1953). Stereochemistry and the Mechanism of Enzymatic Reactions. Biol. Rev..

[24] Vallee B.L., Stein E.A., Sumerwell W.N., Fischer E.H. (1959). Metal Content of α-Amylases of Various Origins. J. Biol. Chem..

[25] Wu H., Tian X., Dong Z., Zhang Y., Huang L., Liu X., Jin P., Lu F., Wang Z. (2018). Engineering of *Bacillus amyloliquefaciens* α-Amylase with Improved Calcium Independence and Catalytic Efficiency by Error-Prone PCR. Starch-Stärke.

[26] Brás N.F., Santos-Martins D., Fernandes P.A., Ramos M.J. (2018). Mechanistic Pathway on Human α-Glucosidase Maltase-Glucoamylase Unveiled by QM/MM Calculations. J. Phys. Chem. B.

[27] Davies G., Henrissat B. (1995). Structures and Mechanisms of Glycosyl Hydrolases. Structure.

[28] MacGregor E.A., Janecek S., Svensson B. (2001). Relationship of Sequence and Structure to Specificity in the Alpha-Amylase Family of Enzymes. Biochim. Biophys. Acta.

[29] Liu P., Ma L., Duan W., Gao W., Fang Y., Guo L., Yuan C., Wu Z., Cui B. (2023). Maltogenic Amylase: Its Structure, Molecular Modification, and Effects on Starch and Starch-Based Products. Carbohydr. Polym..

[30] Zechel D.L., Withers S.G. (2000). Glycosidase Mechanisms:  Anatomy of a Finely Tuned Catalyst. Acc. Chem. Res..

[31] Kelly R.M., Leemhuis H., Dijkhuizen L. (2007). Conversion of a Cyclodextrin Glucanotransferase into an α-Amylase: Assessment of Directed Evolution Strategies. Biochemistry.

[32] Yang S.-J., Min B.-C., Kim Y.-W., Jang S.-M., Lee B.-H., Park K.-H. (2007). Changes in the Catalytic Properties of *Pyrococcus furiosus* Thermostable Amylase by Mutagenesis of the Substrate Binding Sites. Appl. Environ. Microbiol..

[33] Butterworth P.J., Warren F.J., Ellis P.R. (2011). Human α-Amylase and Starch Digestion: An Interesting Marriage. Starch-Stärke.

[34] Ju L., Pan Z., Zhang H., Li Q., Liang J., Deng G., Yu M., Long H. (2019). New Insights into the Origin and Evolution of α-Amylase Genes in Green Plants. Sci. Rep..

[35] Ali Z., Abdullah M., Yasin M.T., Amanat K., Sultan M., Rahim A., Sarwar F. (2025). Recent Trends in Production and Potential Applications of Microbial Amylases: A Comprehensive Review. Protein Expr. Purif..

[36] Westers L., Westers H., Quax W.J. (2004). *Bacillus subtilis* as Cell Factory for Pharmaceutical Proteins: A Biotechnological Approach to Optimize the Host Organism. Biochim. Biophys. Acta (BBA) -Mol. Cell Res..

[37] Silva T.P., de Albuquerque F.S., dos Santos C.W.V., Franco M., Caetano L.C., Pereira H.J.V. (2018). Production, Purification, Characterization and Application of a New Halotolerant and Thermostable Endoglucanase of *Botrytis Ricini* URM 5627. Bioresour. Technol..

[38] Siddiqui K.S., Feller G., D’Amico S., Gerday C., Giaquinto L., Cavicchioli R. (2005). The Active Site Is the Least Stable Structure in the Unfolding Pathway of a Multidomain Cold-Adapted α-Amylase. J. Bacteriol..

[39] Feller G., d’Amic D., Gerday C. (1999). Thermodynamic Stability of a Cold-Active α-Amylase from the Antarctic Bacterium Alteromonas Haloplanctis. Biochemistry.

[40] Rahardjo Y.S.P., Weber F.J., Haemers S., Tramper J., Rinzema A. (2005). Aerial Mycelia of *Aspergillus oryzae* Accelerate α-Amylase Production in a Model Solid-State Fermentation System. Enzym. Microb. Technol..

[41] Kunamneni A., Permaul K., Singh S. (2005). Amylase Production in Solid State Fermentation by the Thermophilic Fungus *Thermomyces lanuginosus*. J. Biosci. Bioeng..

[42] Martău G.-A., Unger P., Schneider R., Venus J., Vodnar D.C., López-Gómez J.P. (2021). Integration of Solid State and Submerged Fermentations for the Valorization of Organic Municipal Solid Waste. J. Fungi.

[43] Gangadharan D., Sivaramakrishnan S., Nampoothiri K.M., Sukumaran R.K., Pandey A. (2008). Response Surface Methodology for the Optimization of Alpha Amylase Production by *Bacillus amyloliquefaciens*. Bioresour. Technol..

[44] Francis F., Sabu A., Nampoothiri K.M., Ramachandran S., Ghosh S., Szakacs G., Pandey A. (2003). Use of Response Surface Methodology for Optimizing Process Parameters for the Production of α-Amylase by *Aspergillus oryzae*. Biochem. Eng. J..

[45] Singhania R.R., Patel A.K., Soccol C.R., Pandey A. (2009). Recent Advances in Solid-State Fermentation. Biochem. Eng. J..

[46] El-Sayed A.K.A., Abou-Dobara M.I., El-Fallal A.A., Omar N.F. (2019). Heterologous Expression, Purification, Immobilization and Characterization of Recombinant α-Amylase AmyLa from *Laceyella* Sp. DS3. Int. J. Biol. Macromol..

[47] Solingen P., Meijer D., Kleij W., Barnett C., Bolle R., Power S., Jones B. (2001). Cloning and Expression of an Endocellulase Gene from a Novel Streptomycete Isolated from an East African Soda Lake. Extremophiles.

[48] Rao D., Wang C., Li X., Shen W., Liu Q., Li Z., Pi S., Han Z., Yang J. (2026). Synergistic Strategies for High Production of *Geobacillus stearothermophilus* α-Amylase in *Bacillus subtilis*. J. Ind. Microbiol. Biotechnol..

[49] Markošová K., Weignerová L., Rosenberg M., Křen V., Rebroš M. (2015). Upscale of Recombinant α-L-Rhamnosidase Production by Pichia Pastoris MutS Strain. Front. Microbiol..

[50] Parashar D., Satyanarayana T. (2016). A Chimeric α-Amylase Engineered from *Bacillus acidicola* and G *Eobacillus thermoleovorans* with Improved Thermostability and Catalytic Efficiency. J. Ind. Microbiol. Biotechnol..

[51] Parashar D., Satyanarayana T. (2017). Production of Chimeric Acidic α-Amylase by the Recombinant Pichia Pastoris and Its Applications. Front. Microbiol..

[52] Deng C., Huang T., Jiang Z., Lv X., Liu L., Chen J., Du G. (2019). Enzyme Engineering and Industrial Bioprocess. Current Developments in Biotechnology and Bioengineering.

[53] Al-Amri A., Al-Ghamdi M.A., Khan J.A., Altayeb H.N., Alsulami H., Sajjad M., Baothman O.A., Nadeem M.S. (2022). Escherichia Coli Expression and Characterization of α-Amylase from *Geobacillus thermodenitrificans* DSM-465. Braz. J. Biol..

[54] Naidu K., Maseko S., Kruger G., Lin J. (2020). Purification and Characterization of α-Amylase from *Paenibacillus* Sp. D9 and *Escherichia coli* Recombinants. Biocatal. Biotransformation.

[55] Slavić M.Š., Kojić M., Margetić A., Stanisavljević N., Gardijan L., Božić N., Vujčić Z. (2023). Highly Stable and Versatile α-Amylase from *Anoxybacillus Vranjensis* ST4 Suitable for Various Applications. Int. J. Biol. Macromol..

[56] Chen J., Chen X., Dai J., Xie G., Yan L., Lu L., Chen J. (2015). Cloning, Enhanced Expression and Characterization of an α-Amylase Gene from a Wild Strain in B. Subtilis WB800. Int. J. Biol. Macromol..

[57] Kholikov A., Vokhidov K., Murtozoyev A., Tóth Z.S., Nagy G.N., Vértessy B.G., Makhsumkhanov A. (2025). Characterization of a Thermostable α-Amylase from *Bacillus licheniformis* 104.K for Industrial Applications. Microorganisms.

[58] Chen Y., Xin Q., Pan L., Wang B. (2023). Improved Recombinant Expression of Maltogenic α-Amylase AmyM in *Bacillus subtilis* by Optimizing Its Secretion and NADPH Production. Fermentation.

[59] Li X., Wang Y., Park J.-T., Gu L., Li D. (2018). An Extremely Thermostable Maltogenic Amylase from Staphylothermus Marinus: Bacillus Expression of the Gene and Its Application in Genistin Glycosylation. Int. J. Biol. Macromol..

[60] Zhang K., Tan R., Yao D., Su L., Xia Y., Wu J. (2021). Enhanced Production of Soluble *Pyrococcus furiosus* α-Amylase in *Bacillus subtilis* through Chaperone Co-Expression, Heat Treatment and Fermentation Optimization. J. Microbiol. Biotechnol..

[61] Ma Y., Shen W., Chen X., Liu L., Zhou Z., Xu F., Yang H. (2016). Significantly Enhancing Recombinant Alkaline Amylase Production in *Bacillus subtilis* by Integration of a Novel Mutagenesis-Screening Strategy with Systems-Level Fermentation Optimization. J. Biol. Eng..

[62] Yang H., Liu L., Shin H., Chen R.R., Li J., Du G., Chen J. (2013). Comparative Analysis of Heterologous Expression, Biochemical Characterization Optimal Production of an Alkaline A-amylase from Alkaliphilic *Alkalimonas amylolytica* in *Escherichia coli* and *Pichia pastoris*. Biotechnol. Progress..

[63] Zhoukun L., Jiale W., Ting W., Wenwen Z., Yan Q., Yan H., Zhongli C. (2018). Efficient Production and Characterization of Maltohexaose-Forming α-Amylase AmyM Secreted From the Methylotrophic Yeast *Pichia pastoris*. Starch Stärke.

[64] Trabelsi S., Sahnoun M., Elgharbi F., Ameri R., Ben Mabrouk S., Mezghani M., Hmida-Sayari A., Bejar S. (2019). *Aspergillus oryzae* S2 AmyA Amylase Expression in Pichia Pastoris: Production, Purification and Novel Properties. Mol. Biol. Rep..

[65] Lin Z., Hu T., Lin L., Li T., Yan Y., Wei W., Wei D. (2025). α-Amylase From *Penicillium Brevicompactum*: Enzymatic Properties and Saccharification. Starch-Stärke.

[66] Cao H., Van Heel A.J., Ahmed H., Mols M., Kuipers O.P. (2017). Cell Surface Engineering of *Bacillus subtilis* Improves Production Yields of Heterologously Expressed α-Amylases. Microb. Cell Fact..

[67] Geissler A.S., Fehler A.O., Poulsen L.D., González-Tortuero E., Kallehauge T.B., Alkan F., Anthon C., Seemann S.E., Rasmussen M.D., Breüner A. (2023). CRISPRi Screen for Enhancing Heterologous α-Amylase Yield in *Bacillus subtilis*. J. Ind. Microbiol. Biotechnol..

[68] Siddiqui K.S., Ertan H., Poljak A., Bridge W.J. (2022). Evaluating Enzymatic Productivity—The Missing Link to Enzyme Utility. Int. J. Mol. Sci..

[69] Yuan S., Li R., Lin B., Yan R., Ye X. (2023). Engineering of *Bacillus amyloliquefaciens* α-Amylase for Improved Catalytic Efficiency by Error-Prone PCR. Starch Stärke.

[70] Cui J.-N., Hu W., Liu Y.-X., Li Y.-L., Hu J.-H., Liu Z.-Y., Chen J.-H. (2023). Isolation and Screening of High-Yielding α-Amylase Mutants of *Bacillus subtilis* by Heavy Ion Mutagenesis. Appl. Biochem. Biotechnol..

[71] Richardson T.H., Tan X., Frey G., Callen W., Cabell M., Lam D., Macomber J., Short J.M., Robertson D.E., Miller C. (2002). A Novel, High Performance Enzyme for Starch Liquefaction: DISCOVERY AND OPTIMIZATION OF a LOW pH, THERMOSTABLE α-AMYLASE. J. Biol. Chem..

[72] Zhang J., Li X., Wang M., Ren S., Li M., Liu Y., Lu F., Li Q., Li Y. (2025). Rational Construction of a Robust *Bacillus amyloliquefaciens* Cell Factory for Acid-Stable α Amylase Production. J. Agric. Food Chem..

[73] Liu Y., Lu F., Li Y., Wang J., Gao C. (2008). Acid Stabilization of *Bacillus licheniformis* Alpha Amylase through Introduction of Mutations. Appl. Microbiol. Biotechnol..

[74] Yao D., Su L., Li N., Wu J. (2019). Enhanced Extracellular Expression of *Bacillus stearothermophilus* α-Amylase in *Bacillus subtilis* through Signal Peptide Optimization, Chaperone Overexpression and α-Amylase Mutant Selection. Microb. Cell Fact..

[75] Niu C., Zhang D., Zou D., Zhao W., Liu D., Huang K., Wei X., Li D., Ye C., Xiong H. (2025). Novel Insights for α-Amylase Improvement: Leveraging Amylopectin into Accurate Molecular Docking and Mutant Selection. J. Agric. Food Chem..

[76] Prakash O., Jaiswal N. (2010). α-Amylase: An Ideal Representative of Thermostable Enzymes. Appl. Biochem. Biotechnol..

[77] Pan S., Gu Z., Ding N., Zhang Z., Chen D., Li C., Hong Y., Cheng L., Li Z. (2019). Calcium and Sodium Ions Synergistically Enhance the Thermostability of a Maltooligosaccharide-Forming Amylase from *Bacillus stearothermophilus* STB04. Food Chem..

[78] Ban X., Dhoble A.S., Li C., Zhang Y., Gu Z., Cheng L., Hong Y., Li Z. (2017). Potassium and Sodium Ions Enhance the Activity and Thermostability of 1,4-α-Glucan Branching Enzyme from *Geobacillus thermoglucosidasius* in the Presence of Glycerol. Int. J. Biol. Macromol..

[79] Bharwad K., Shekh S., Singh N.K., Patel A., Joshi C. (2023). Heterologous Expression and Biochemical Characterization of Novel Multifunctional Thermostable α-Amylase from Hot-Spring Metagenome. Int. J. Biol. Macromol..

[80] Syed D.G., Agasar D., Pandey A. (2009). Production and Partial Purification of α-Amylase from a Novel Isolate *Streptomyces gulbargensis*. J. Ind. Microbiol. Biotechnol..

[81] Siddiqui K.S., Poljak A., Guilhaus M., Feller G., D’Amico S., Gerday C., Cavicchioli R. (2005). Role of Disulfide Bridges in the Activity and Stability of a Cold-Active α-Amylase. J. Bacteriol..

[82] Abedi E., Torabizadeh H., Orden L. (2023). Enhancement of Alpha-Amylase’s Stability and Catalytic Efficiency After Modifying Enzyme Structure Using Calcium and Ultrasound. Food Bioprocess. Technol..

[83] Musawi A., Salih M. (2025). The Impact of Low-Level Laser Irradiation on the Activity of Alpha-Amylase. Photonics.

[84] Zhang X., Qin W., Tian X., Huang M. (2011). Effect of Microwave Irradiation on Secondary Structure of α-Amylase by Circular Dichroism. J. Cent. South. Univ. Technol..

[85] Saqib A., Saif-ur-Rehman, Ali H., Hassan N., Ali A., Rashid M.H. (2025). Gama Rays Mediated Improvement of Catalytic Efficiency and Thermostability of Glucoamylase by Replacing Active Site Leucine to Isoleucene from Super Koji (*Aspergillus oryzae*). PLoS ONE.

[86] Smolin N., Winter R. (2006). A Molecular Dynamics Simulation of SNase and Its Hydration Shell at High Temperature and High Pressure. Biochim. Biophys. Acta (BBA) -Proteins Proteom..

[87] Beltagy E.A., Abouelwafa A., Barakat K.M. (2022). Bioethanol Production from Immobilized Amylase Produced by Marine *Aspergillus flavus* AUMC10636. Egypt. J. Aquat. Res..

[88] Hwang E.T., Gu M.B. (2013). Enzyme Stabilization by Nano/Microsized Hybrid Materials. Eng. Life Sci..

[89] Rodrigues R.C., Barbosa O., Ortiz C., Berenguer-Murcia Á., Torres R., Fernandez-Lafuente R. (2014). Amination of Enzymes to Improve Biocatalyst Performance: Coupling Genetic Modification and Physicochemical Tools. RSC Adv..

[90] Stepankova V., Bidmanova S., Koudelakova T., Prokop Z., Chaloupkova R., Damborsky J. (2013). Strategies for Stabilization of Enzymes in Organic Solvents. ACS Catal..

[91] Ashly P.C., Joseph M.J., Mohanan P.V. (2011). Activity of Diastase α-Amylase Immobilized on Polyanilines (PANIs). Food Chem..

[92] Razzaghi M., Homaei A., Hemmati R., Saberi D., Kavousipour S. (2023). Cellulose-Gold Nanohybrid as an Effective Support to Enhance the Catalytic Efficiency and Stability of α-Amylase from *Bacillus aquimaris*. J. Mol. Liq..

[93] Akbulut K., Taranacı S., Özkök S., Varan N.E., Yildirim D., Binay B. (2025). Heterologous Expression of Calcium-Independent Mesophilic α-Amylase from *Priestia Megaterium*: Immobilization on Genipin-Modified Multi-Walled Carbon Nanotubes and Silica Supports to Enhance Thermostability and Catalytic Activity. Bioorganic Chem..

[94] Iqbal M.N., Jaworski A., Pinon A.C., Bengtsson T., Hedin N. (2023). Activity and Stability of Nanoconfined Alpha-Amylase in Mesoporous Silica. ACS Mater. Au.

[95] Yandri Y., Tiarsa E.R., Suhartati T., Satria H., Irawan B., Hadi S. (2022). The Stability Improvement of α-Amylase Enzyme from *Aspergillus Fumigatus* by Immobilization on a Bentonite Matrix. Biochem. Res. Int..

[96] Atiroğlu V., Atiroğlu A., Özacar M. (2021). Immobilization of α-Amylase Enzyme on a Protein @metal–Organic Framework Nanocomposite: A New Strategy to Develop the Reusability and Stability of the Enzyme. Food Chem..

[97] Defaei M., Taheri-Kafrani A., Miroliaei M., Yaghmaei P. (2018). Improvement of Stability and Reusability of α-Amylase Immobilized on Naringin Functionalized Magnetic Nanoparticles: A Robust Nanobiocatalyst. Int. J. Biol. Macromol..

[98] Desai R.P., Dave D., Suthar S.A., Shah S., Ruparelia N., Kikani B.A. (2021). Immobilization of α-Amylase on GO-Magnetite Nanoparticles for the Production of High Maltose Containing Syrup. Int. J. Biol. Macromol..

[99] Siddiqui K.S. (2015). Some like It Hot, Some like It Cold: Temperature Dependent Biotechnological Applications and Improvements in Extremophilic Enzymes. Biotechnol. Adv..

[100] Movahedpour A., Asadi M., Khatami S.H., Taheri-Anganeh M., Adelipour M., Shabaninejad Z., Ahmadi N., Irajie C., Mousavi P. (2022). A Brief Overview on the Application and Sources of A-amylase and Expression Hosts Properties in Order to Production of Recombinant A-amylase. Biotech. App Biochem..

[101] Yuan S., Yan R., Lin B., Li R., Ye X. (2023). Improving Thermostability of *Bacillus amyloliquefaciens* Alpha-Amylase by Multipoint Mutations. Biochem. Biophys. Res. Commun..

[102] Li S., Yang Q., Tang B. (2020). Improving the Thermostability and Acid Resistance of *Rhizopus oryzae* A-amylase by Using Multiple Sequence Alignment Based Site-directed Mutagenesis. Biotech. App Biochem..

[103] Liu J., Han L., Li J., Du G., Zhang G. (2025). Modification of Flexible Regions for Enhanced Thermal Stability of Alkaline Amylase. J. Agric. Food Chem..

[104] Höcker B., Jürgens C., Wilmanns M., Sterner R. (2001). Stability, Catalytic Versatility and Evolution of the (Βα)8-Barrel Fold. Curr. Opin. Biotechnol..

[105] Cui X., Yuan X., Li S., Hu X., Zhao J., Zhang G. (2022). Simultaneously Improving the Specific Activity and Thermostability of α-Amylase BLA by Rational Design. Bioprocess. Biosyst. Eng..

[106] Wang C.-H., Lu L.-H., Huang C., He B.-F., Huang R.-B. (2020). Simultaneously Improved Thermostability and Hydrolytic Pattern of Alpha-Amylase by Engineering Central Beta Strands of TIM Barrel. Appl. Biochem. Biotechnol..

[107] Zhu M., Zhai W., Jiang H., Lin L., Wei W., Wei D. (2023). Exploiting Loop-Grafting Strategy Resorting on Computer-Aided Design to Improve the Thermostability of Alpha-Amylase from *Geobacillus stearothermophilus*. Process Biochem..

[108] Fehler A.O., Kallehauge T.B., Geissler A.S., González-Tortuero E., Seemann S.E., Gorodkin J., Vinther J. (2022). Flagella Disruption in *Bacillus subtilis* Increases Amylase Production Yield. Microb. Cell Fact..

[109] Yao D., Han X., Gao H., Wang B., Fang Z., Li H., Fang W., Xiao Y. (2023). Enhanced Extracellular Production of Raw Starch-Degrading α-Amylase in *Bacillus subtilis* through Expression Regulatory Element Modification and Fermentation Optimization. Microb. Cell Fact..

[110] Zhang J., Xu X., Li X., Chen X., Zhou C., Liu Y., Li Y., Lu F. (2021). Reducing the Cell Lysis to Enhance Yield of Acid-Stable Alpha Amylase by Deletion of Multiple Peptidoglycan Hydrolase-Related Genes in *Bacillus amyloliquefaciens*. Int. J. Biol. Macromol..

[111] Hernández-Heredia S., Peña-Castro J.M., Aguilar-Uscanga M.G., Olvera C., Nolasco-Hipólito C., Del Moral S. (2022). AmyJ33, a Truncated Amylase with Improved Catalytic Properties. Biotechnol. Lett..

[112] Liu L., Deng Z., Yang H., Li J., Shin H., Chen R.R., Du G., Chen J. (2014). In Silico Rational Design and Systems Engineering of Disulfide Bridges in the Catalytic Domain of an Alkaline α-Amylase from Alkalimonas Amylolytica To Improve Thermostability. Appl. Environ. Microbiol..

[113] Wang Y., Li C., Ban X., Gu Z., Hong Y., Cheng L., Li Z. (2022). Disulfide Bond Engineering for Enhancing the Thermostability of the Maltotetraose-Forming Amylase from Pseudomonas Saccharophila STB07. Foods.

[114] Rebholz G.F., Sebald K., Dirndorfer S., Dawid C., Hofmann T., Scherf K.A. (2021). Impact of Exogenous α-Amylases on Sugar Formation in Straight Dough Wheat Bread. Eur. Food Res. Technol..

[115] Farooq M.A., Ali S., Hassan A., Tahir H.M., Mumtaz S., Mumtaz S. (2021). Biosynthesis and Industrial Applications of α-Amylase: A Review. Arch. Microbiol..

[116] Cho I.H., Peterson D.G. (2010). Chemistry of Bread Aroma: A Review. Food Sci. Biotechnol..

[117] Bae W., Lee S.H., Yoo S., Lee S. (2014). Utilization of a Maltotetraose-Producing Amylase as a Whole Wheat Bread Improver: Dough Rheology and Baking Performance. J. Food Sci..

[118] Yang T., Zhong L., Jiang G., Liu L., Wang P., Zhong Y., Yue Q., Ouyang L., Zhang A., Li Z. (2022). Comparative Study on Bread Quality and Starch Digestibility of Normal and Waxy Wheat (*Triticum aestivum* L.) Modified by Maltohexaose Producing α-Amylases. Food Res. Int..

[119] Suriya J., Bharathiraja S., Krishnan M., Manivasagan P., Kim S.-K. (2016). Marine Microbial Amylases. Advances in Food and Nutrition Research.

[120] Parashar D., Satyanarayana T. (2018). An Insight Into Ameliorating Production, Catalytic Efficiency, Thermostability and Starch Saccharification of Acid-Stable α-Amylases From Acidophiles. Front. Bioeng. Biotechnol..

[121] Yuansah S.C., Laga A. (2023). Pirman Enzymatic Saccharification of Purple Sweet Potato Flour by α-Amylase, Xylanase, Mannanase and Amyloglucosidase for Liquid Sugar Production. IOP Conf. Ser. Earth Environ. Sci..

[122] Lee M.-E., Shin H.-Y., Bhardwaj N., Cho B.-H., Hwang D.-H., Jeong W.-Y., Han S.O. (2023). Effective Bioconversion of Fungal-Spoiled Starchy Food Waste into Fermentable Sugars Using Fungi-Degrading, Artificial Amylosomes. Bioresour. Technol..

[123] He M., Jin Y., Liu M., Yang G., Zhou R., Zhao J., Wu C. (2023). Metaproteomic Investigation of Enzyme Profile in Daqu Used for the Production of Nongxiangxing Baijiu. Int. J. Food Microbiol..

[124] Xia Y., Zhu M., Du Y., Wu Z., Gomi K., Zhang W. (2022). Metaproteomics Reveals Protein Composition of Multiple Saccharifying Enzymes in Nongxiangxing Daqu and Jiangxiangxing Daqu under Different Thermophilic Temperatures. Int. J. Food Sci. Technol..

[125] Fan W., Qian M.C. (2006). Characterization of Aroma Compounds of Chinese “Wuliangye” and “Jiannanchun” Liquors by Aroma Extract Dilution Analysis. J. Agric. Food Chem..

[126] Pires E.J., Teixeira J.A., Brányik T., Vicente A.A. (2014). Yeast: The Soul of Beer’s Aroma—A Review of Flavour-Active Esters and Higher Alcohols Produced by the Brewing Yeast. Appl. Microbiol. Biotechnol..

[127] Parés Viader R., Yde M.S.H., Hartvig J.W., Pagenstecher M., Carlsen J.B., Christensen T.B., Andersen M.L. (2021). Optimization of Beer Brewing by Monitoring α-Amylase and β-Amylase Activities during Mashing. Beverages.

[128] Goesaert H., Slade L., Levine H., Delcour J.A. (2009). Amylases and Bread Firming–an Integrated View. J. Cereal Sci..

[129] Souza P.M.d., Magalhães P.D.O.E. (2010). Application of Microbial α-Amylase in Industry-a Review. Braz. J. Microbiol..

[130] Olempska-Beer Z.S., Merker R.I., Ditto M.D., DiNovi M.J. (2006). Food-Processing Enzymes from Recombinant Microorganisms—A Review. Regul. Toxicol. Pharmacol..

[131] Sewalt V., Shanahan D., Gregg L., La Marta J., Carrillo R. (2016). The Generally Recognized as Safe (GRAS) Process for Industrial Microbial Enzymes. Ind. Biotechnol..

